# Double deep reinforcement learning twin-delayed agents for performance improvement of a grid-connected wave energy conversion system

**DOI:** 10.1038/s41598-026-55262-w

**Published:** 2026-05-27

**Authors:** Faisal Aldawsari, Ahmed Mahdy, Ziad M. Ali, Ahmed F. Zobaa, Shady H. E. Abdel Aleem, Hany M. Hasanien

**Affiliations:** 1 Electrical Engineering Department, College of Engineering at Wadi Addawaser, Prince Sattam bin Abdulaziz University, 11991 Wadi Addawaser, Saudi Arabia; 2 Electrical Power and Machines Department, Faculty of Engineering, Ain Shams University, Cairo, 11517 Egypt; 3 Department of Engineering, College of Engineering, Design and Physical Sciences, Brunel University of London, Uxbridge, UB8 3PH UK; 4 Basic Sciences Council, Academy of Scientific Research and Technology, Cairo, 11516 Egypt; 5 Departament of Electrical Engineering, Badya University, Giza, 12566 Egypt; 6 Department of Electrical Engineering, College of Engineering, University of Sharjah, Sharjah, 27272 United Arab Emirates

**Keywords:** Archimedes wave swing, Deep learning, Power system control, Twin-delayed deep deterministic policy gradient, Wave energy conversion systems, Energy science and technology, Engineering, Mathematics and computing

## Abstract

This study introduces a novel approach using two deep learning agents, trained with the twin-delayed deep deterministic policy gradient (TD3) algorithm, to replace the PI controllers used for the control of grid-connected Archimedes Wave Swing (AWS) wave energy conversion systems. The generator converter’s controller has two mandatory objectives: minimizing losses in the stator and maximizing energy extraction from incident sea waves. These goals are achieved by controlling the generator’s *dq* currents using a TD3 agent on the rectifier side. In addition, the grid-side inverter’s controller is responsible for regulating both the DC link and the point-of-common-coupling voltages. In the new configuration, two approaches are proposed in this work: either a single deep learning agent replaces the four proportional-integral (PI) controllers on the inverter side, or a hybrid approach combining two PI controllers with a TD3 agent. To verify the reliability of the TD3 agents, the system is analyzed in both steady and transient states under fault conditions. Furthermore, the TD3 agents’ performance is benchmarked against the classical PI controller configuration in MATLAB Simulink. The results demonstrate better dynamic and steady-state responses from the hybrid-TD3 agent on the grid side than from the full PI classical configuration.

## Introduction

Ocean wave energy, a resource with immense global potential, reaching up to 32,000 TWh/yr, has attracted considerable interest recently^1^. Unlike solar and wind, wave energy offers greater predictability and availability. This makes it a strong candidate for integration into modern power systems. By 2035, the levelized cost of electricity generated from good wave energy resources could drop under 70 €/MWh^2^. However, wave energy converters (WECs) have several drawbacks, including maximizing energy extraction from waves, economic feasibility (high costs), and grid integration. This work focuses on addressing the problem of successful grid connection and surviving grid faults. In the literature, numerous wave energy converters, including the AWS, oscillating water column (OWC), Wave Dragon, and Pelamis, have been controlled and optimized to maximize energy extraction and ensure strong grid connection. For instance, a wave-to-grid (W2G) architecture has been developed for a point absorber wave energy converter to enhance energy extraction and provide a smooth power supply to the grid^3^. The mechanical variables are estimated using an extended Kalman filter, enabling sensorless control of the device. Moreover, these variables are employed as input to the model-predictive control (MPC) technique for controlling the generator side. In addition, a battery energy storage system is used to provide smooth power to the power grid. Another study addressed the problem of peak-to-average power in wave energy converters^4^. Strong, instantaneous sea waves can cause an extreme burst of power (peak) for a short period. This requires an oversized generator and turbine to handle this power for a short time, leading to a higher cost of the wave energy converter. The authors deploy a high-speed stop valve in series with the OWC air turbine to restrict air flow during periods of high power. The results confirmed the viability of the control method and achieved a low peak-to-average power ratio. However, the disadvantage is the violation of the constraints for a very short period. Furthermore, a study focuses on maximizing energy extraction from AWS devices by regulating the *q*-axis current of the generator-side to control the generator velocity^5^. The system uses lithium-ion batteries as an energy storage element to exchange reactive power with the AWS device as needed, thereby eliminating the need for the grid to reverse power. Moreover, a study proposes a W2G architecture for a floating wave energy converter that uses a LiTe-Con controller to enhance wave energy extraction^6^. An ultra-capacitor is integrated into the DC link to provide the reactive power required by the wave energy converter. Both generator-side and grid-side converters employ Lyapunov-based nonlinear controllers to minimize copper losses and current errors, achieve a unity power factor, and regulate the DC link voltage. Additionally, recent research has focused on applying advanced optimization algorithms and exploring new energy vectors to enhance the performance of renewable energy systems. For example, the Red-Tailed Hawk optimization algorithm has been applied for optimal PV array reconfiguration under partial shading conditions, significantly improving energy yield in practical applications such as irrigation systems^7^. Furthermore, the potential of green hydrogen as a complementary energy carrier for storing surplus renewable energy has been extensively investigated, with studies evaluating regional opportunities and challenges for large-scale hydrogen production using solar resources^8^. Although these studies focus on photovoltaic systems and hydrogen production, their core concepts are highly relevant to wave energy systems. In the context of the AWS devices, the PI controllers were tuned using the Coot optimization algorithm to improve the dynamic stability of the grid-connected AWS system under severe fault conditions^9^. Furthermore, recent advances in control theory have enabled the successful application of fractional-order and nonlinear PID-based controllers across various industrial domains. For instance, a study in^10^ demonstrated the effectiveness of a multi-stage FOPI controller for nonlinear motor systems, while^11^ proposed a hyperbolic tangent-based PID for pressure control. Although these studies focus on different applications, they highlight the growing interest in developing robust controllers for nonlinear, uncertain systems like the AWS converter. For instance, a study focused on adopting the fractional-order PID (FOPID) for controlling the AWS system^12^. The FOPID controllers are tuned using the hybrid jellyfish search and particle swarm optimization, achieving higher performance than PI controllers. In^13^, a hydraulic energy-storage wave energy converter temporarily stores energy harvested from waves in high-pressure accumulators. At a certain pressure, the converter starts the generation stage and provides power to the grid. When the pressure goes down below a certain threshold, the generator stops and returns to the energy storage phase. The system incorporates batteries to further smooth the power given to the grid. In^14^, five PI controllers are employed to manage the rectifier and inverter in an AWS converter. In addition to the coupling of a distributed battery energy storage (DBES) with a DC/DC converter integrated to the DC link to smooth the power provided to the electrical grid. The same control configuration can be found in^15^, where a supercapacitor energy storage is employed instead of the DBES to smooth the output power. Furthermore, a study proposes a hybrid energy storage system comprising a supercapacitor for short-term, fast response and batteries for long-term, slow response to smooth the injected power from a wave energy park composed of multiple wave energy converters^16^. Moreover, a study proposes a low-voltage ride-through controller that increases reactive power injection during fault conditions for AWS systems^17^. The magnitude of the reactive power injection is dependent on the dip in the voltage at the point of common coupling ${V}_{PCC}$. Moreover, the generator’s active power is set to zero, and a braking chopper is used to prevent overvoltage in the DC link.

The primary focus of this paper is on the AWS, as it is a widely used wave energy converter^18^. In this study, the main objective is to implement a novel control methodology that hasn’t been explored within the context of AWS technology. Recent AI-driven developments have opened new avenues for revolutionary control methods in renewable energy systems. Deep learning agents demonstrate strong capabilities for handling nonlinear, time-varying, and uncertain dynamics that align with the operational environment of wave energy converters. Moreover, deep learning approaches can directly learn optimal control policies by acquiring data through actions and rewards received during interaction with the environment. There are various deep learning algorithms for training an agent, including deep Q-learning (DQN), soft actor-critic (SAC), proximal policy optimization (PPO), twin-delayed deep deterministic policy gradient (TD3), and deep deterministic policy gradient (DDPG), among others. For instance, an RL-beta method is adopted for maximum power point tracking (MPPT) in a solar energy system^19^. Beta is calculated from voltage and current measurements, which the agent uses to produce a discrete action: a change in the duty cycle. Moreover, a study uses the TD3 algorithm to train an agent controlling a multi-level inverter in a solar energy system^20^. The TD3 agent is employed to minimize the errors in the *dq* currents by selecting the optimal *dq* voltages of a solar inverter. Another work uses the DQN to train an agent to directly select the optimal switching action for a three-level inverter^21^. The results are compared with the conventional MPC strategy. In addition, a study uses the dandelion optimizer with either DDPG or PPO for MPPT in a 100 MW PV system^22^. The dandelion optimizer provides the reference voltage for MPPT. The PPO or DDPG is used to minimize the error between the reference and the actual system voltages by selecting the optimal duty cycle for pulse-width modulation (PWM) of a boost converter in the PV system. Furthermore, a study proposes a physics-guided deep learning framework in place of conventional lookup tables for real-time wind farm flow control under time-varying conditions^23^. This approach utilizes a clustering-based precomputation strategy that cuts computational overhead by 85.5% while achieving 40% better control performance than standard methods. In addition, a paper focuses on solving the voltage fluctuations in a multi-feeder distribution system using deep learning agents^24^. The system configuration consists of a main agent responsible for controlling the tap position of the on-load tap changer and sub-agents for adjusting the reactive power of each inverter in the associated PV systems. The results indicate a voltage deviation of only 1.28% from the optimum. Moreover, a deep learning framework combined with MPC is proposed for energy management in a building^25^. The MPC employs an approximate model of the system to filter and ensure that the agent’s action remains within the constraints. For wave energy converters, the researchers focus on maximizing energy extraction from waves. For instance, DQN is employed for a point absorber WEC^26^. The agent takes an action by changing the load resistance to maximize energy extraction. Furthermore, DDPG was applied in AWS devices to maximize energy extraction by changing the reference quadrature-axis current (${i}_{q-ref}$) to vary the generator force under changing sea states^27^. This controller was compared with multiple energy maximization strategies, and the results confirm its greater energy extraction capability. In addition, many studies use the SAC algorithm to control the power-take-off (PTO) force to increase energy harvest^28–30^. In^31^, the oscillating wave surge converter’s energy extraction is maximized by the formulation of an adaptive damping policy under different sea conditions. Several algorithms, including TD3, SAC, and PPO, are employed to achieve this objective.

In this work, the main goal is to replace the classical PI controller configuration with two deep learning agents. These agents minimize the errors in control signals by providing suitable reference *dq* voltages, as explained later. These voltages can take any value between − 1 and 1. This leads to the requirement of a continuous action-type algorithm. In this study, the TD3 is employed due to its numerous advantages over DQN and DDPG. The novel contributions of this work are summarized as follows:

- The TD3 deep learning agent is trained for 10 s to minimize the generator losses and maximize energy extraction by controlling the generator’s *dq* currents. Subsequently, the agent is evaluated for an additional 40 s (unseen states) to verify its reliability.
- The generator-side TD3 agent was compared with the classical configuration of PI controllers and achieved lower ISE for the *dq* currents. Moreover, the agent was subjected to various sea states and to changes in the floater mass to assess its reliability.
- Additionally, another two TD3 agents for the grid-side are trained for 10 s (one under steady-state and the other under transient state).
- The two grid-side agents and the PI controllers are benchmarked against each other under the steady-state for 50 s. In addition, the three configurations are analyzed during the transient state under different grid faults, including three-line (LLL), double-line to ground (2LG), line to ground (LG), three-line to ground (3LG), and line-line (LL) faults, to assess the efficacy of the agents. These results were obtained by simulating the grid-connected AWS system in Simulink.
- The controller efficacy was analyzed under the effect of different grid SCR values (1.5, 3, 5). The controller demonstrated its efficiency in the steady state and during transient operation (SCR = 5), further emphasizing its superior performance.

The remaining sections of this paper are organized as follows: the AWS system model and the linear generator are discussed in Sect. “Modeling of the AWS system”. The classical PI control loop and the new control strategy using double deep learning agents are detailed in Sect. Control of an AWS WEC connected to the grid. Section “TD3 agents’ results under various operating conditions” presents benchmarking of the double-agent configuration against the classical configuration in steady and transient states. The concluding remarks are summarized in Sect. “Conclusions”.

## Modeling of the AWS system

### Modeling of the AWS device

The AWS is a submerged WEC that relies on the reciprocating motion of its floater (up-and-down movement) caused by sea waves. A linear generator converts the AWS motion to usable electrical power. Newton’s second law formulates the equations of motion presented by Eqs. (1) and (2)^32^:

1 $${m}_{f}\frac{dv}{dt} = {F}_{drag}+{F}_{grav}+{F}_{hS}+{F}_{rad}+{F}_{sp}+{F}_{wb}+{F}_{gen}+{F}_{end}+{F}_{e}+ {F}_{bear}$$

2 $$v=\frac{dx}{dt}$$

*x* and *v* are the position and velocity of the AWS floater, respectively. When $${F}_{e}$$ is positive, the floater moves downwards, which corresponds to the positive direction of *x*and *v*. The resistance encountered by the floater during its oscillatory movement is represented by the drag force ($${F}_{drag}$$). This force is presented in Eq. (3) and has two drag coefficients ($${C}_{DUP})$$ and $$\left({C}_{DDW}\right)$$ for both upward and downward motions. In addition, water density and the floater’s outer surface area are symbolized by *ρ* and $${S}_{F}$$. The gravitational force ($${F}_{grav}$$) is articulated by Eq. (4). In this equation, $${m}_{f}$$ and *g* are the floater’s mass and the gravitational acceleration.

3 $${F}_{drag}=\left\{\begin{array}{c}-\frac{1}{2}\rho {S}_{F}v\left|v\right|{C}_{DUP}, v\ge 0\\ -\frac{1}{2}\rho {S}_{F}v\left|v\right|{C}_{DDW}, v<0\end{array}\right.$$

4 $${F}_{grav} = -{m}_{f}g$$

The difference in pressure on the buoy causes the hydrostatic force (${F}_{hS}$). ${F}_{hS}$ is presented in Eq. (5) and consists of multiple parameters, including the tide’s level (*η*), the atmospheric pressure (${p}_{amp}$), the floater height (${h}_{f}$), the inner area of the floater (${S}_{f}$), and the depth at mid-position (${d}_{f}$)^33^.

5 $${F}_{hS} = -{S}_{F}\left(\rho g\left({d}_{f} + \eta - x\right)+ {p}_{amp}\right)+ ({S}_{F} - {S}_{f} )\left(\rho g\right({d}_{f} + \eta + {h}_{f} - x) +{p}_{amp})$$

The radiation of water waves is caused by the oscillatory motion of the floater. This phenomenon is expressed by the radiation force (${F}_{rad}$). ${F}_{rad}$ is presented by Eq. (6). In this equation, the added water mass and the radiation damping kernel are denoted by *R*(*t*) and${ m}_{add}$.

6 $${F}_{rad}=-{m}_{add}\frac{dv}{dt}-{\int }_{0}^{t}R(t-\tau )v\left(\tau \right)d\tau$$

A spring is used to restore the AWS floater to the equilibrium position. The effect of spring is presented by the spring force (${F}_{sp}$) expressed by Eq. (7). ${F}_{sp}$ is dependent on the length at equilibrium (${L}_{eq}$), spring force in the equilibrium state (${F}_{sp-eq}$), the heat capacity rate (*γ*), and the floater’s equilibrium position (${x}_{eq}$).

7 $${{F}_{sp}={F}_{sp-eq}\left(\frac{{L}_{eq}}{{L}_{eq+x-{x}_{eq}}}\right)}^{\gamma }$$

The floater contains water brakes (${F}_{wb}$) with a damping coefficient (${\beta }_{wb}$) that prevent the floater from exceeding *x=4 m*. In addition to the mechanical end stops provided to protect the floater from damage at *x=4.5 m*. The force ${F}_{wb}$ and ${F}_{end}$ are formulated using Eqs. (8) and (9).

8 $${F}_{wb}=-{\beta }_{wb}v\left|v\right|, x\ge 4 or x\le -4$$

9 $${F}_{end}= -\frac{v\left({m}_{add}+{m}_{f}\right)}{0.1}, x\ge 4.5$$

The excitation force of waves that results in the floater motion is symbolized by ${F}_{e}$. This force and its corresponding components are given in Eqs. (10)–(15):

10 $${F}_{e} = \rho g{S}_{F} \eta \left(t\right) {K}_{pw}$$

11 $$\eta \left(t\right) =\sum _{i=1}^{N}\frac{{H}_{i}}{2}\mathrm{sin}({\omega }_{i}t+ {\theta }_{i})$$

12 $${A}_{i} = \frac{{H}_{i}}{2}=\sqrt{2S\left({\omega }_{i}\right){\Delta }{\omega }_{i}}$$

13 $$S\left(\omega \right) = \frac{487 {{H}_{s}}^{2}}{{{T}_{p}}^{4}{\omega }^{5}}{e}^{-\frac{1948.2}{{{T}_{p}}^{4}{\omega }^{4}}}$$

14 $${K}_{pw}=\frac{\mathrm{cosh}\left(k\right(h-d\left) \right)}{\mathrm{cosh}kh}$$

15 $${\omega }^{2}=gk\mathrm{tanh}\left(kh\right)$$

$${F}_{e}$$of irregular sea waves is produced by combining several sinusoidal regular waves (21 waves are selected with angular frequencies between 0.5 and 2.5 rad/s). The irregular waves’ total elevation is denoted by ($$\eta \left(t\right)$$). $${A}_{i}$$, $${\omega }_{i}$$, and $${\theta }_{i}$$represent the amplitude, the angular frequency, and the phase shift of each sinusoidal wave. The Bretschneider spectrum (*S*(*ω*)) is adopted to form irregular sea waves, and the angular frequency difference between these waves is denoted by $${\Delta }{\omega }_{i}$$ with a value of 0.1 rad/Sect.^34^. This spectrum relies on two main parameters, the significant height ($${H}_{s}$$) and the peak energy period ($${T}_{p}$$). Another important parameter is the wave pressure decay factor ($${K}_{pw}$$). The pressure of sea waves is the highest at the surface. However, as depth increases, the effect of this pressure decreases. Hence, $${K}_{p}$$ is required to provide the actual pressure on the floater based on the depth (*d*), the wave number (*k*), and the distance to the seabed from the floater (*h*). Moreover, the wave particles have horizontal wave velocity (*u*) and acceleration ($$\dot{u}$$) that affects the AWS floater in the form of the horizontal force ($${F}_{H}).$$ $${F}_{H}$$contributes to the frictional force $$\left({F}_{bear}\right)$$ that affects the AWS bearings with a friction coefficient (*μ*). $${F}_{H}$$ is dependent on the drag coefficient ($${c}_{D}$$), the inertia coefficient ($${c}_{M}$$), and the outer diameter ($${d}_{out}$$). Finally, $${F}_{H}$$is obtained by performing an integration of the wave particles’ horizontal effect across the floater length. The equations are provided in Eqs. (16)–(21)^35^.

16 $${F}_{bear} = -\mu . \mathrm{s}\mathrm{i}\mathrm{g}\mathrm{n}\left(v\right) \left|{F}_{H}\right|$$

17 $${dF}_{H}(z,t) = {c}_{M}\rho \frac{\pi }{4}{{d}_{out}}^{2}\dot{u}(z,t)dz + {c}_{D}\rho \frac{1}{2}{d}_{out}u(z,t)\left|u(z,t)\right|dz$$

18 $${F}_{H} ={\int }_{z=0}^{{z=h}_{f}}{dF}_{H}(z,t)$$

19 $$u =\frac{H\omega }{2}\frac{\mathrm{cosh}\left(k\right(h-d\left) \right)}{\mathrm{sinh}kh}\mathrm{cos}\left(\omega t\right)$$

20 $$\dot{u}= -\frac{H{\omega }^{2}}{2}\frac{\mathrm{cosh}\left(k\right(h-d\left) \right)}{\mathrm{sinh}kh}\mathrm{sin}\left(\omega t\right)$$

21 $$d ={d}_{f}+{h}_{f}-z$$

### Modeling of the linear generator

The AWS linear generator can be modeled using the conventional *abc* frame or the *dq0* frame. In this work, the *dq0* equations are used to model the generator. In^36^, Feng proposes the equations that represent the generated terminal *dq* voltages (${v}_{d}$and ${v}_{q}$) and currents (${i}_{d}$and ${i}_{q}$) during both positive and negative floater velocities. These formulas are essential for controlling the generator. These voltages are expressed by Eqs. (22) and (23):

22 $${v}_{d} = -R{i}_{d}+ {X}_{s} {i}_{q}- {L}_{s}\left(\frac{{\omega }_{gen}}{\left|{\omega }_{gen}\right|}\right)\left(\frac{d{i}_{d}}{dt}\right)$$

23 $${v}_{q} = -R{i}_{q}- {X}_{s} {i}_{d}- {L}_{s}\left(\frac{{\omega }_{gen}}{\left|{\omega }_{gen}\right|}\right)\left(\frac{d{i}_{q}}{dt}\right)+{\omega }_{gen}{\psi }_{PM}$$

*R* and ${L}_{s}$ symbolize the generator phase resistance and inductance. ${X}_{s}$denotes the phase reactance (${X}_{s}=\left|{\omega }_{gen}\right|{L}_{s}$). ${\omega }_{gen}$ is the generator angular speed (2π*v*/*λ*). *λ* is the pole width. ${\psi }_{PM}$ represents the permanent magnets’ flux linkage. The generator output power (${P}_{gen }$) and the force (${F}_{gen }$) are linked together using Eq. (24).

24 $${F}_{gen }=\frac{-{P}_{gen }}{v}=\frac{-3{\omega }_{gen}{i}_{q}{\psi }_{PM}}{2v}=\frac{-3\pi {i}_{q}{\psi }_{PM}}{\lambda }$$

The model parameters adopted for the AWS system are presented in Table 1.

**Table 1:** Numerical values of the AWS system parameters.

Symbol	Value	Symbol	Value	Symbol	Value
${m}_{f}$	4×${10}^{5}$ kg	γ	1.4	${\psi }_{PM}$	23 Wb
${m}_{add}$	3.55×${10}^{5}$ kg	${S}_{F}$	95 ${\mathrm{m}}^{2}$	*λ*	0.1 m
*ρ*	1025 kg/${\mathrm{m}}^{3}$	*σ*	4 m	${C}_{DDW}$	0.4
${p}_{amp}$	1×${10}^{5}$N/${\mathrm{m}}^{2}$	*µ*	0.1	${C}_{DUP}$	0.2
${\beta }_{wb}$	1.5×${10}^{6}$ kg/m	*η*	0	*R*	0.29 Ω
*N*	21 waves	${c}_{M}$	2	${L}_{s}$	0.031 H
${d}_{f}$	11 m	${S}_{f}$	79 ${\mathrm{m}}^{2}$	${h}_{f}$	28.5 m
${c}_{D}$	1	*g*	9.8 m/${\mathrm{s}}^{2}$	${d}_{out}$	11 m

## Control of an AWS WEC connected to the grid

**Fig. 1:**
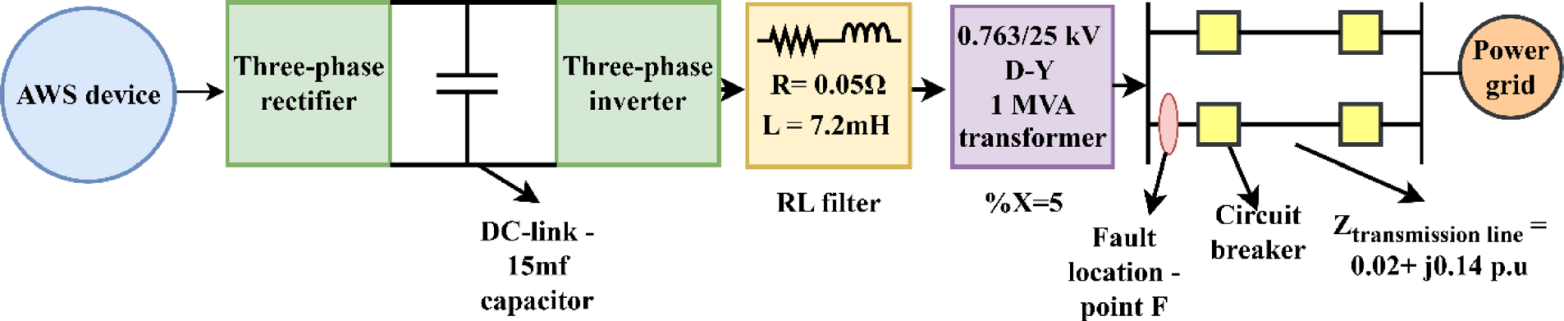
The overall grid-connected architecture of the AWS device.

The linear generator outputs a three-phase voltage characterized by fluctuating magnitude and frequency. This voltage can’t be provided directly to the power grid without some modifications first. Therefore, a combined configuration of a rectifier, a DC link, and an inverter is adopted to improve suitability. The inverter is followed by a simple RL filter to eliminate a significant portion of switching harmonics. The filter output is fed to a step-up transformer to raise the voltage to 25 kV, suitable for the medium voltage distribution network. The transformer is connected to the grid via double transmission lines. Figure 1 depicts the system’s architecture.

### The mathematical formulation of the twin-delayed deep deterministic policy gradient algorithm (TD3)

TD3 is considered the upgraded version of the DDPG algorithm^37^. Both are used for continuous action spaces and employ an actor-critic architecture, which is appropriate for the control system. The classical DDPG algorithm uses one actor and one critic:

- The actor is the final neural network that provides the optimum action based on current observations.
- The critic estimates the quality of the action (*Q*-value) taken by the actor during the training process and hence controls the update procedure of the actor’s neural network weights and biases.

The common problem with the DDPG’s critic is the overestimation of the *Q*-value, or in other words, the critic’s problem of being too optimistic, and the training instability. These problems are addressed in the TD3 as follows:

- Regarding the first problem, the TD3 adopts two critics (twin) instead of just one. During target value computation, the minimum of the two critics’ values is used (clipped double *Q*-learning).
- With respect to the second issue, in DDPG, the actor is updated every step. However, TD3 introduces a delay in the update process (updates the actor once every two steps), which makes the training more stable.
- DDPG can have unstable updates due to errors in *Q*-values. Therefore, TD3 adds noise to the target action to make the policy more robust. It smooths the *Q*-value estimate around the target action.

The training process starts with the random initialization of the weights and biases of the actor (${\pi }_{\theta }$) and the two critics (${Q}_{\phi 1}$ and ${Q}_{\phi 2}$) neural networks. In addition to copying the neural networks of the actor and the two critics to form the target networks ${\pi }_{{\theta }^{{\prime }}}$, ${Q}_{{\phi 1}^{{\prime }}}$, and ${Q}_{{\phi 2}^{{\prime }}}$. *π* and *Q* signify the actor policy and the *Q*-value provided by the critic network, respectively. Moreover, *θ*, *ϕ*, ${\theta }^{{\prime }}$, and ${\phi }^{{\prime }}$ are the parameters of the actor, critic, target actor, and target critic networks, respectively. The actor’s current policy takes an action with noise (${ \epsilon }_{explore}$) added for the exploration process during environment interaction presented by Eq. (25):

25 $$a={\pi }_{\theta }\left(s\right)+{\epsilon}_{explore}$$

where *a*, *s*, and *ε* denote the action taken, current state, and the exploration noise. After *a*, the agent obtains a reward (*r*) and goes to a new state (${s}^{{\prime }}$). This information (*s*, *a*, *r*, and ${s}^{{\prime }}$) is collected in the experience replay buffer ($\mathbb{D}$) for updating the neural networks. The agent keeps taking actions and collecting experiences until the replay buffer reaches its minimum size (a mini-batch). To train the neural networks, a random mini-batch is taken from the replay buffer. Then, the target action (${a}^{{\prime }}$) is computed with noise (${ \epsilon}_{smooth}$) for smoothing the *Q*-function using Eq. (26), and the target *Q*-value (*y*) is computed via Eq. (27):

26 $${a}^{{\prime }}={\pi }_{{\theta }^{{\prime }}}\left({s}^{{\prime }}\right)+{\epsilon}_{smooth}$$

27 $$y=r+(1-d)\gamma \underset{i=\mathrm{1,2}}{{min}}{Q}_{{\phi }_{i}^{{\prime }}}\left({s}^{{\prime }},{a}^{{\prime }}\right)$$

In Eq. (27), *γ* denotes the discount factor, which represents the weight provided for the future rewards and how much we care about those rewards in comparison with the instant reward from taking an action, and *d* presents the termination flag. The next step is updating the twin critic networks using the mean squared error between the predicted *Q*-value (${Q}_{{\phi }_{i}}(s,a)$) and the target value (*y*). This is given by the loss function ($\mathcal{L}\left({\phi }_{i}\right)$) in Eq. (28):

28 $$\mathcal{L}\left({\phi }_{i}\right)={E}_{\left(s,a,r,{s}^{{\prime }},d\right)\sim \mathbb{D}}\left[{\left({Q}_{{\phi }_{i}}\left(s,a\right)-y\right)}^{2}\right]$$

where *E* is the expectation (average over one batch). To update the critic network, a gradient descent for $\mathcal{L}\left({\phi }_{i}\right)$ with respect to the network parameters (${\phi }_{i}$) is performed in Eq. (29):

29 $$\mathcal{L}{\nabla }_{{\phi }_{i}}\mathcal{L}\left({\phi }_{i}\right)=E\left[2\left({Q}_{{\phi }_{i}}\left(s,a\right)-y\right){\nabla }_{{\phi }_{i}}{Q}_{{\phi }_{i}}\left(s,a\right)\right]$$

Update of the network parameters is achieved using a learning rate (${\alpha }_{Q}$) in Eq. (30):

30 $${\phi }_{i}\leftarrow {\phi }_{i}-{\alpha }_{Q}{\nabla }_{{\phi }_{i}}\mathcal{L}\left({\phi }_{i}\right)$$

The actor is updated every couple of steps using the policy gradient in Eq. (31). In this equation, the weights are updated in the direction that maximizes the expected reward by the critic (gradient ascent). TD3 uses only one critic.

31 $${\nabla }_{\theta }J\left(\theta \right)={E}_{s\sim \mathbb{D}}\left[{\nabla }_{a}{Q}_{{\phi }_{1}}\left(s,a\right){\left.\right|}_{a={\pi }_{\theta }\left(s\right)}{\nabla }_{\theta }{\pi }_{\theta }\left(s\right)\right]$$

Like the critic, the actor network parameters are updated using a learning rate (${\alpha }_{a}$) as given in Eq. (32):

32 $$\theta \leftarrow \theta +{\alpha }_{a}{\nabla }_{\theta }J\left(\theta \right)$$

The critic and actor target networks are updated slowly every two steps using Eqs. (33) and (34):

33 $${\phi }_{i}^{{\prime }}\leftarrow \tau {\phi }_{i}+\left(1-\tau \right){\phi }_{i}^{{\prime }}$$

34 $${\theta }^{{\prime }}\leftarrow \tau \theta +\left(1-\tau \right){\theta }^{{\prime }}$$

where *τ* is the target network update rate. This completes the overall explanation of TD3. In the next part, the application of the TD3 agent to the rectifier and the inverter is discussed.

### Control of the three-phase rectifier using PI controllers

The generator-side two-level rectifier comprises six IGBT switches. This converter’s controller has two objectives. The minimization of losses by controlling ${i}_{d}$ and maintaining it at zero (${i}_{d-ref}=0$), and the maximization of energy extraction by controlling ${i}_{q}$ at a certain value dependent on *v*. A simple technique, approximate current control, is adopted. The reference current (${i}_{q-ref}$) is given in Eq. (35)^38^:

35 $${I}_{q-ref} =138v$$

**Fig. 2:**
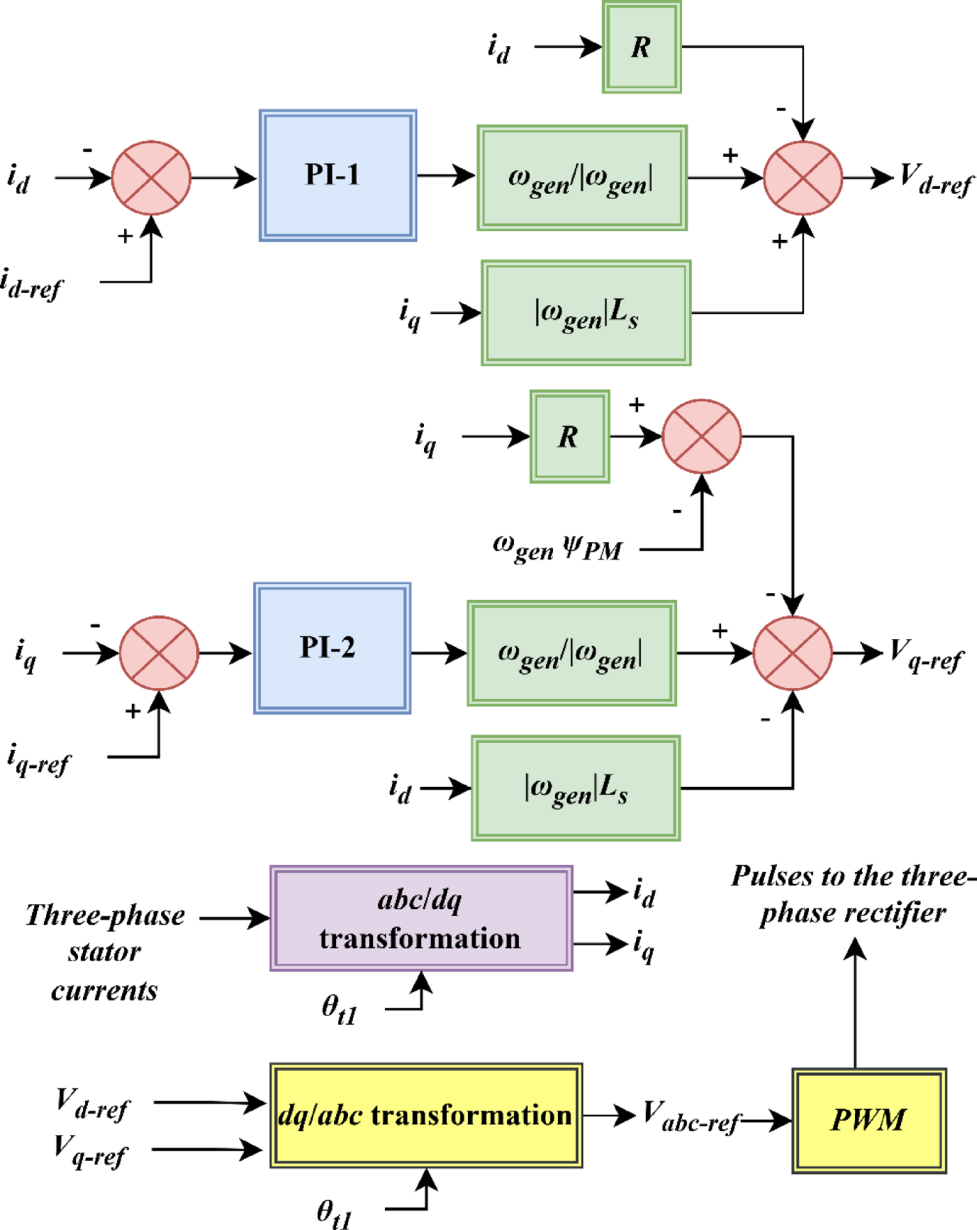
Control loop of the generator side using PI controllers.

The 138 value was selected by trial and error^38^. The control loop of these two currents is designed based on Eqs. (22) and (23). The control loop is formed of two PI controllers, to which the *dq* currents’ errors (${e}_{id}$ and ${e}_{iq}$) are provided. The PI controllers generate the appropriate *dq* reference voltages. These voltages are converted into *abc* voltages (${V}_{abc-ref}$) using the inverse Clarke-Park transformation. The transformation angle (${\theta }_{t1}$) is calculated using ($\frac{2{\uppi }x}{\lambda } -\frac{{\uppi }}{2}$). Finally, the switching actions are provided to the IGBTs by comparing ${V}_{abc-ref}$ with a 5-kHz triangular waveform. The control loop is depicted in Fig. 2.

### Control of the three-phase rectifier using a TD3 agent

**Fig. 3:**
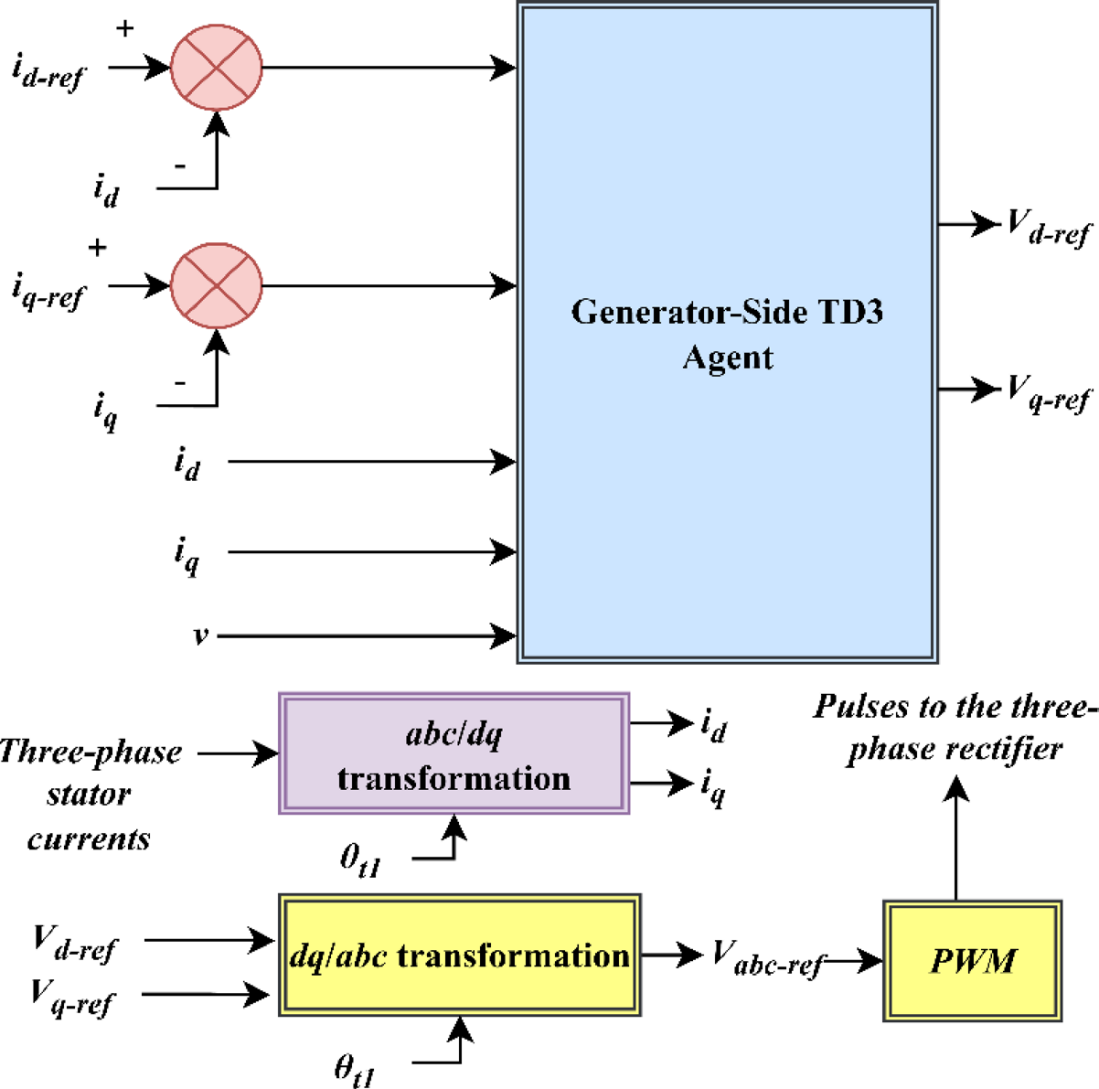
Control loop of the generator side using the TD3 agent.

The same objectives for the *dq* currents are accomplished via the generator-side TD3 agent. This agent provides the reference *dq* voltages based on some appropriate observations. The selected inputs upon which the agent takes decisions are the *dq* currents’ errors (${e}_{id}$ and ${e}_{iq}$), the values of the *dq* currents, and *v* (as the direction of motion affects the control action). Subsequently, the agent would provide the reference *dq* voltages that are converted into *abc* voltages (${V}_{abc-ref}$) using the inverse Clarke-Park transformation. The transformation angle (${\theta }_{t1}$) is calculated using ($\frac{2{\uppi }x}{\lambda } -\frac{{\uppi }}{2}$). Finally, the switching actions are provided to the IGBTs by comparing ${V}_{abc-ref}$ with a 5-kHz triangular waveform. The control loop is depicted in Fig. 3.

Also, a reward function (${r}_{gen}$) based on the minimization of the *dq* currents errors is formulated using Eqs. (36)–(38) to guide the agent in the training:

36 $${r}_{id}=0.03-\left|{e}_{id}\right|$$

37 $${r}_{iq}=0.03-\left|{e}_{iq}\right|$$

38 $${r}_{gen}={r}_{id}+{r}_{iq}$$

**Fig. 4:**
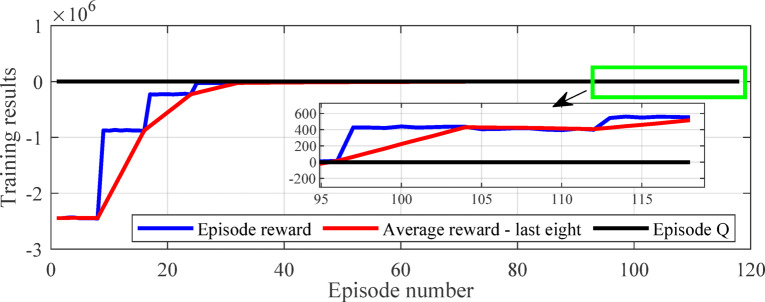
Results of training the generator-side TD3 agent.

Moreover, the most important training parameters are given in Table 2. These values are selected after many trial-and-error sessions. The agent is trained in MATLAB Simulink for 10 s. The results of the training procedure are illustrated in Fig. 4.

**Table 2:** The generator-side agent training options.

Parameter	Value
Actor network	2 hidden layers with 64 and 128 neurons (ReLU function) with an output layer (tanh function).
Critic network	2 hidden layers with 128 and 256 neurons (ReLU function).
Agent sample time	${1\times 10}^{-4}$
${\alpha }_{a}$	${1\times 10}^{-5}$
${\alpha }_{Q}$	${1\times 10}^{-3}$
${\upgamma }$	0.99
Mini-batch size	1024
Experience buffer length	${3\times 10}^{6}$
*τ*	0.005
Policy update frequency	2
Gaussian noise - action taken	Standard deviation = 0.5, and decay rate = ${1\times 10}^{-6}$
Gaussian noise - target action	Standard deviation = 0.2
Parallelization	8 parallel workers - async mode.
Wall-clock training time	3 h
Total environment steps	${12.8\times 10}^{6}$ in 128 episodes

### Control of the three-phase inverter using PI controllers

**Fig. 5:**
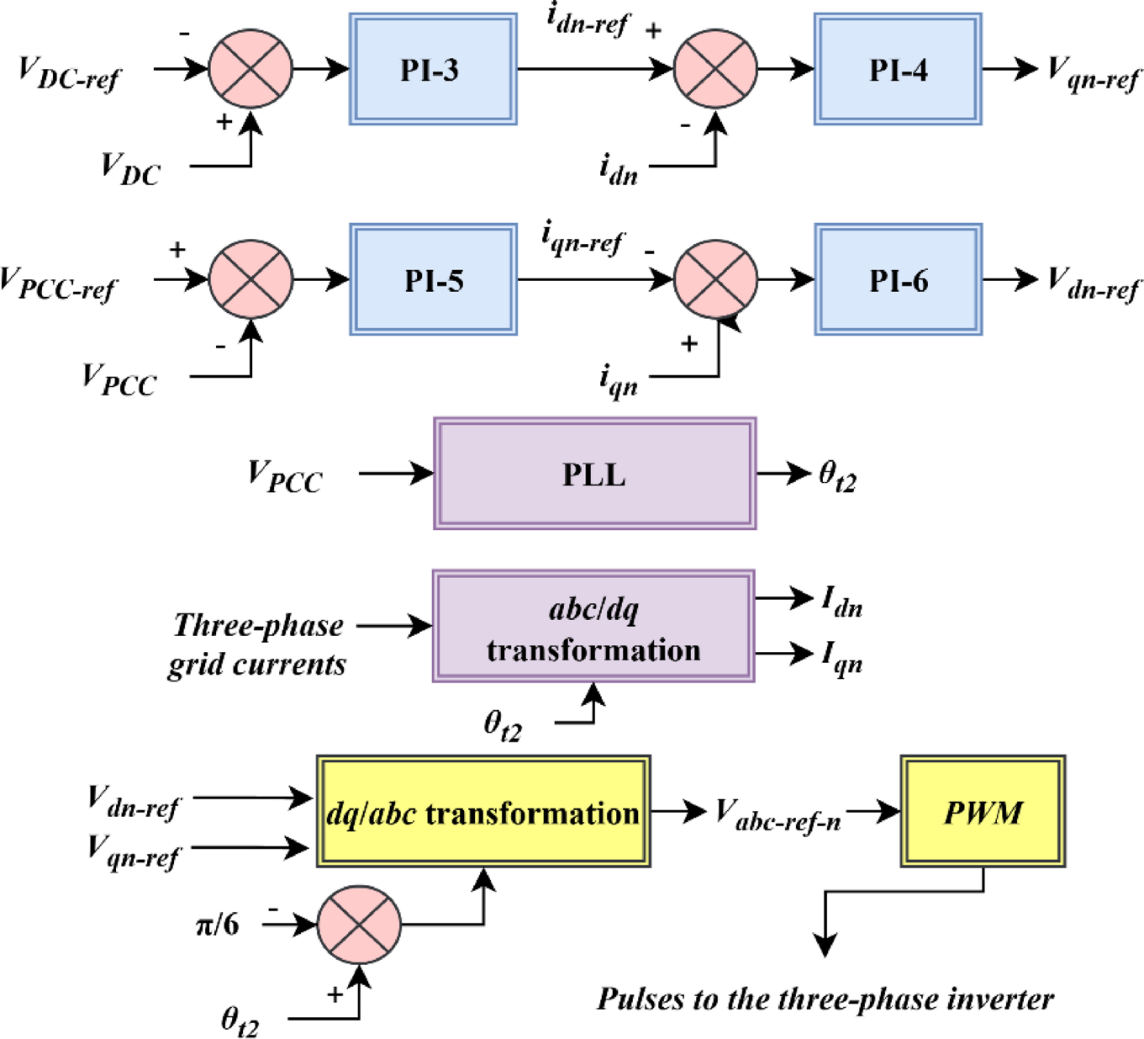
Control loop of the two-level inverter.

The controller regulates ${V}_{DC}$ and ${V}_{PCC}$ at 1 pu. This is achieved by controlling the grid-side *dq* currents (${i}_{dn}$& ${i}_{qn}$). These currents are responsible for adjusting the grid’s active and reactive powers (${P}_{grid}$and${Q}_{grid}$), and hence, ${V}_{DC}$ and ${V}_{PCC}$ could be controlled. The classical control system is formed of four PI controllers. The first controller’s input is the error in ${V}_{DC}$ (${e}_{{V}_{DC}}$) which in turn gives the reference direct axis current (${i}_{dn-ref}$). The error between ${i}_{dn-ref}$and ${i}_{dn}$ is given to another controller that gives the reference quadrature axis voltage ${(V}_{qn-\mathrm{r}\mathrm{e}\mathrm{f}}$). Similarly, the error in ${V}_{PCC}$ (${e}_{{V}_{PCC}}$) is provided to another controller that generates the reference quadrature axis current (${i}_{qn-ref}$). The error between ${i}_{qn-ref}$and ${i}_{qn}$ is an input to another controller that gives the reference direct axis voltage ${(V}_{dn-\mathrm{r}\mathrm{e}\mathrm{f}}$). ${V}_{dn-\mathrm{r}\mathrm{e}\mathrm{f}}$ and ${V}_{qn-\mathrm{r}\mathrm{e}\mathrm{f}}$ are converted into *abc* voltages ${(V}_{abc-ref-n}$) using the inverse Clarke-Park transformation. The transformation angle (${\theta }_{t2}$) is calculated using a phase-locked loop (PLL). $30^\circ$ is subtracted from ${\theta }_{t2}$ due to different connections of the primary and the secondary windings of the transformer. Finally, the switching actions are provided to the IGBTs by comparing ${V}_{abc-ref-n}$ with a 1-kHz triangular waveform. A summary of the control loop of the inverter is depicted in Fig. 5. Each PI controller has two gains (${k}_{p}$and${ k}_{i}$). The gains are presented by *G=*[${k}_{p1}{ k}_{i1}$ ${k}_{p2}{ k}_{i2}$ ….], where the proportional and integral gains of the *i*-th PI controller are denoted by ${k}_{pi}$and ${k}_{ii}$, respectively. These gains were tuned in^38^ and *G=*[627.69 717.1 800 1000 7.71 80 1.2 22.5 3.66 219.3 2.2 25].

### Control of the three-phase inverter using a TD3 agent

**Fig. 6:**
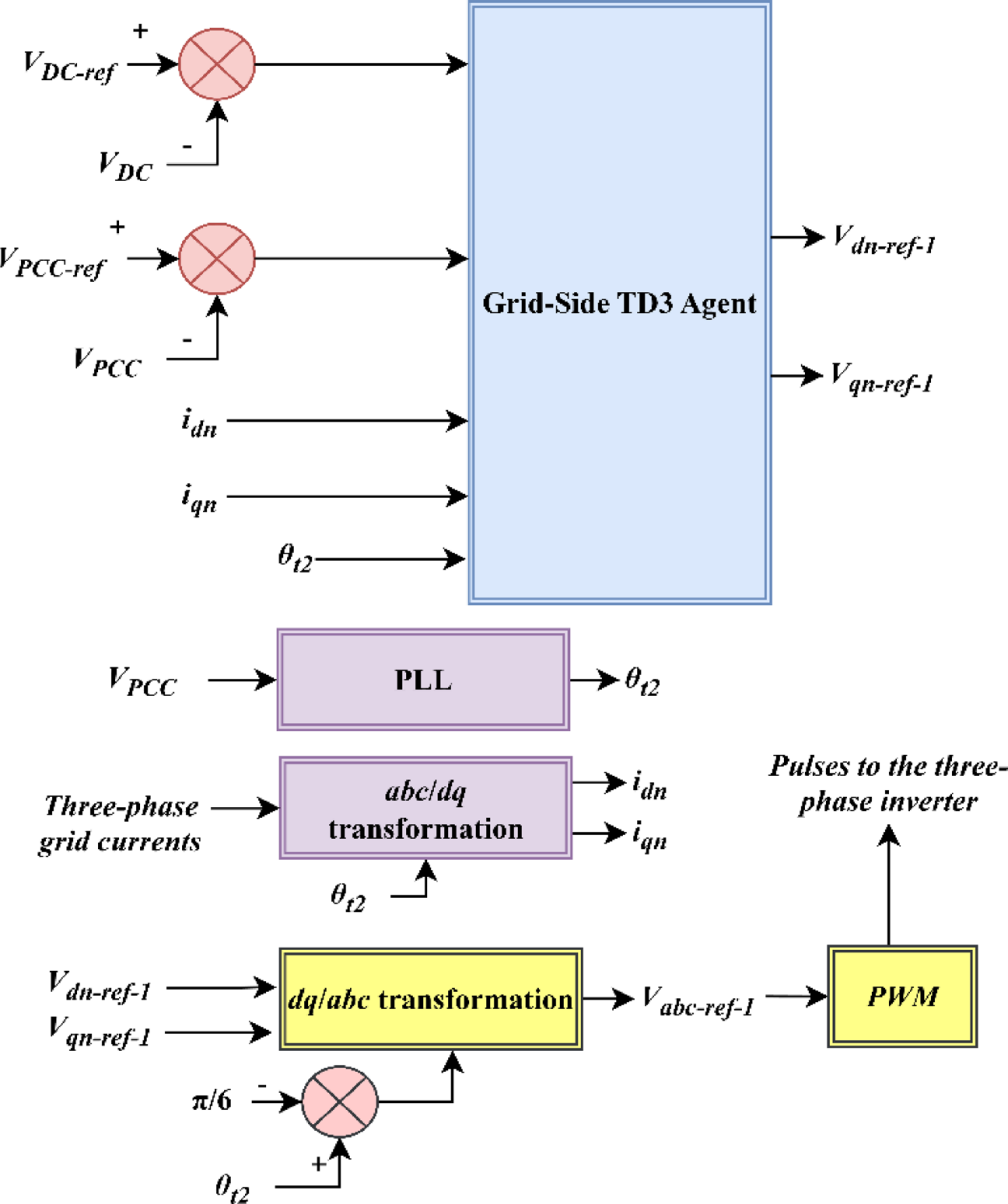
New control loop of the grid side using the TD3 agent.

There are two TD3-agent approaches proposed for the grid-side controller. The first one is the replacement of the four PI controllers with a TD3 agent that takes several observations as an input and provides the reference grid-side *dq* voltages (${V}_{dn-\mathrm{r}\mathrm{e}\mathrm{f}-1}$and ${V}_{qn-\mathrm{r}\mathrm{e}\mathrm{f}-1}$). The selected inputs are ${e}_{{V}_{DC}}$, ${e}_{{V}_{PCC}}$, ${i}_{dn}$, ${i}_{qn}$, and ${\theta }_{t2}$. The new control loop is illustrated in Fig. 6.

The reward function (${r}_{grid-voltages}$) selected for training this agent is given in Eqs. (39)–(41):

39 $${r}_{{V}_{DC}}=0.005-\left|{e}_{{V}_{DC}}\right|$$

40 $${r}_{{V}_{PCC}}=0.005-\left|{e}_{{V}_{PCC}}\right|$$

41 $${r}_{grid-voltages}=5({r}_{{V}_{DC}}+{r}_{{V}_{PCC}})$$

**Fig. 7:**
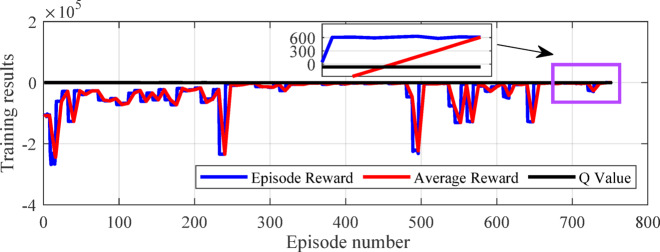
Results of training the grid-side TD3 agent.

The agent is trained for 10 s under steady state. The training parameters are given in Table 3, and the training results are shown in Fig. 7.

**Table 3:** The grid-side agent training options.

Parameter	Value
Actor network	2 hidden layers with 32 and 64 neurons (ReLU) with an output layer (tanh).
Critic network	2 hidden layers with 64 and 64 neurons (ReLU).
Agent sample time	${4\times 10}^{-4}$
${\alpha }_{a}$	${5\times 10}^{-6}$
${\alpha }_{Q}$	${1\times 10}^{-5}$
${\upgamma }$	0.995
Experience buffer length	${2\times 10}^{6}$
Mini-batch size	1024
*τ*	0.005
Policy update frequency	2
Gaussian noise - action taken	Standard deviation = 0.2 and decay rate = ${1\times 10}^{-5}$
Gaussian noise - target action	Standard deviation = 0.2
Parallelization	8 parallel workers - async mode.
Wall-clock training time	16.5 h
Total environment steps	${18.8\times 10}^{6}$ in 752 episodes

**Fig. 8:**
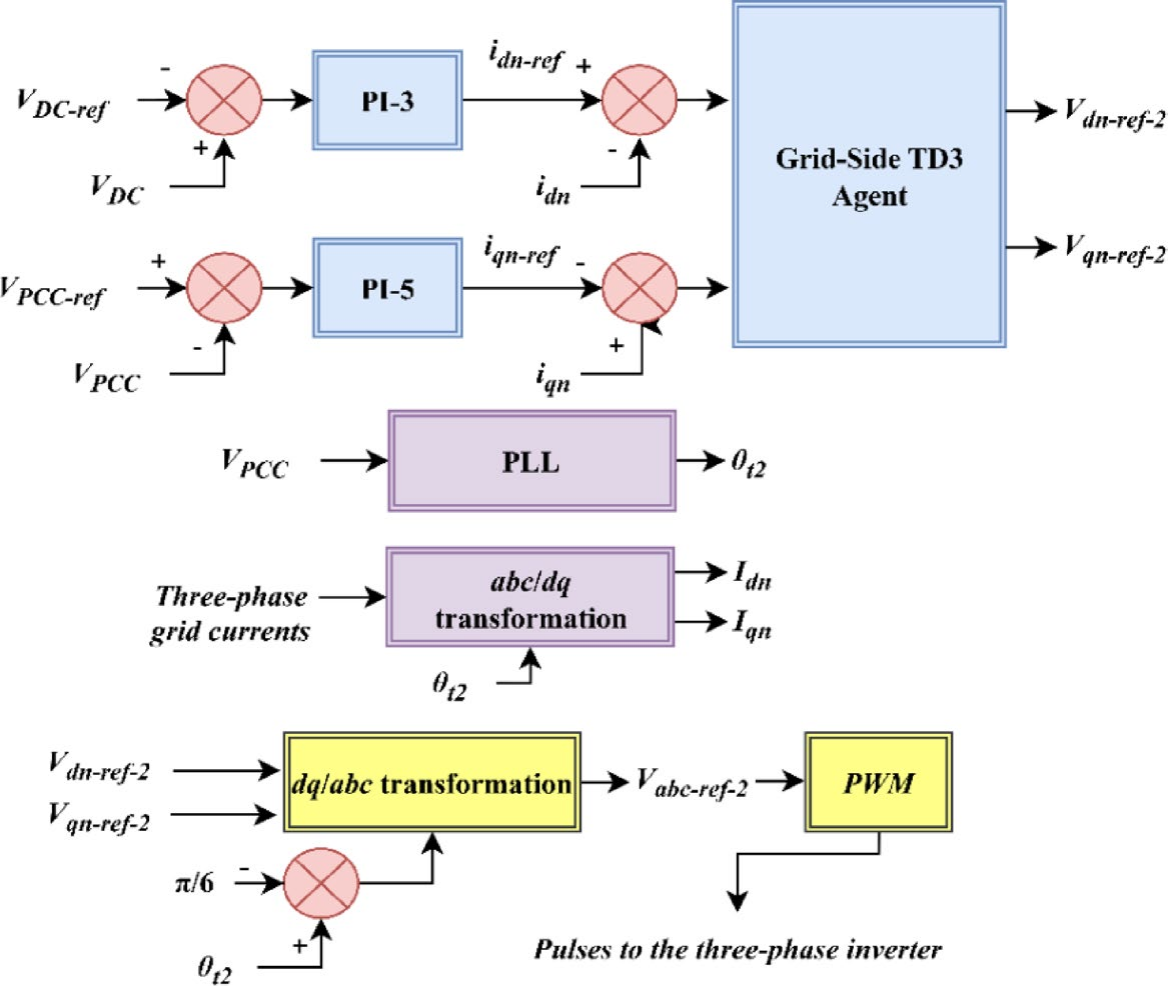
The hybrid PI-TD3 agent control loop.

In the second approach, a hybrid PI-TD3 agent is deployed. In this control strategy, the first two PI controllers that provide the reference grid *dq* currents are kept as they are, and the second pair of PI controllers is exchanged with a TD3 agent. This control loop is given in Fig. 8. The agent’s control objective is to minimize the errors in *dq* currents. Therefore, the reward function (${r}_{grid-currents}$) for training this agent is proposed in Eqs. (42)–(44):

42 $${r}_{{i}_{dn}}=0.01-\left|{e}_{{i}_{dn}}\right|$$

43 $${r}_{{i}_{qn}}=0.01-\left|{e}_{{i}_{qn}}\right|$$

44 $${r}_{grid-currents}=5({r}_{{i}_{dn}}+{r}_{{i}_{qn}})$$

**Fig. 9:**
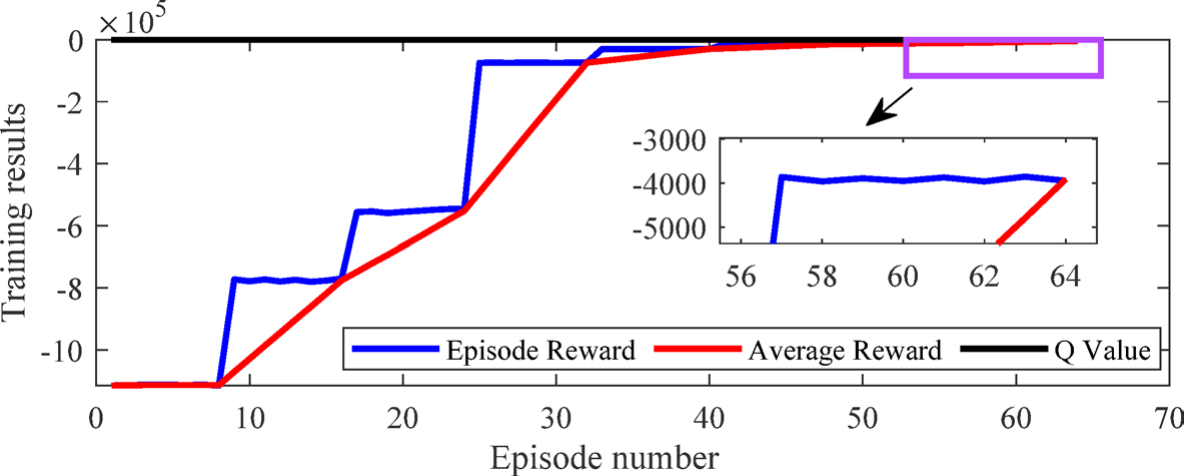
Results of training the grid-side hybrid PI-TD3 agent.

The agent is trained for 10 s under the 3LG condition to help the actor acquire a strong policy that suppresses the *dq* currents during fault. The training parameters are given in Table 4, and the training results are shown in Fig. 9.

**Table 4:** The hybrid PI-TD3 agent training options.

Parameter	Value
Actor network	2 hidden layers with 18 and 32 neurons (ReLU) with an output layer (tanh).
Critic network	2 hidden layers with 64 and 128 neurons (ReLU).
Agent sample time	${1\times 10}^{-4}$
${\alpha }_{a}$	${1\times 10}^{-5}$
${\alpha }_{Q}$	${1\times 10}^{-4}$
${\upgamma }$	0.995
Experience buffer length	${2\times 10}^{6}$
Mini-batch size	1024
*τ*	0.005
Policy update frequency	2
Parallelization	8 parallel workers - async mode.
Gaussian noise - action taken	Standard deviation = 0.2 and decay rate = ${1\times 10}^{-6}$
Gaussian noise - target action	Standard deviation = 0.2
Wall-clock training time	1.5 h
Total environment steps	${6.4\times 10}^{6}$ in 64 episodes

The summary of these agents, including observations, actions, and the corresponding reward functions, is provided in Table 5.

**Table 5:** Summary of the agents.

Agent	Observations vector	Actions vector	Reward function
Generator-side TD3	$[{e}_{id},{e}_{iq} {i}_{d},{i}_{q},v]$	$[{V}_{d-\mathrm{r}\mathrm{e}\mathrm{f}}, {V}_{q-\mathrm{r}\mathrm{e}\mathrm{f}}]$	${r}_{gen}={r}_{id}+{r}_{iq}$
PI-TD3	${[e}_{{V}_{DC}}$, ${e}_{{V}_{PCC}}$, ${i}_{dn}$, ${i}_{qn}$, ${\theta }_{t2}]$	$[{V}_{dn-\mathrm{r}\mathrm{e}\mathrm{f}-1}, {V}_{qn-\mathrm{r}\mathrm{e}\mathrm{f}-1}]$	${r}_{grid-voltages}=5({r}_{{V}_{DC}}+{r}_{{V}_{PCC}})$
Hybrid PI-TD3	${[e}_{{i}_{dn}}$, ${e}_{{i}_{qn}}]$	$[{V}_{dn-\mathrm{r}\mathrm{e}\mathrm{f}-2},{V}_{qn-\mathrm{r}\mathrm{e}\mathrm{f}-2}]$	${r}_{grid-currents}=5({r}_{{i}_{dn}}+{r}_{{i}_{qn}})$

In the next section, these controllers are investigated under steady state (untrained states) and transient state (fault conditions).

## TD3 agents’ results under various operating conditions

In this part, the double-agent configuration is analyzed during steady and transient states under various grid faults. The system is executed in MATLAB/Simulink 2024b with a fixed step solver type ode4 and a solver time step of 10 µs. The converters use non-ideal IGBT/diode switches with an on-state resistance of 1 mΩ.

### Generator-side agent performance

**Fig. 10:**
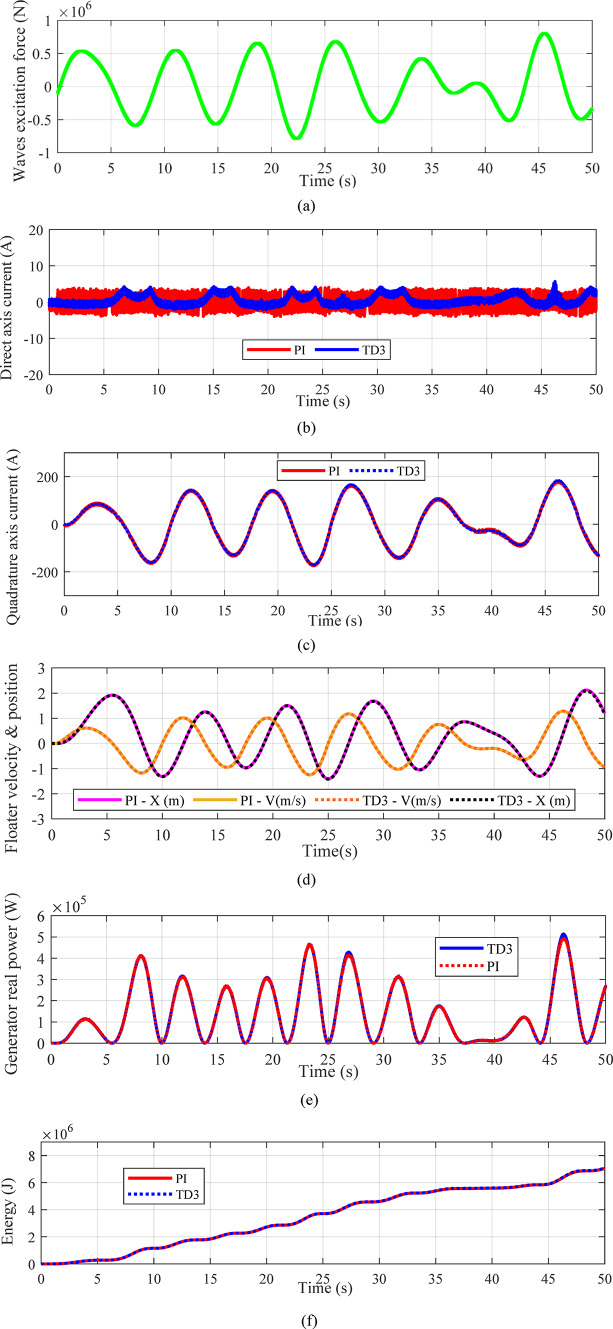
The PI controllers and the TD3 agent performance under irregular waves characterized by ${H}_{s}= 4 m$ and ${T}_{p}=8 \mathrm{s}$, (**a**) ${F}_{e}$, (**b**) ${i}_{d}$, (**c**) ${i}_{q}$, (**d**) *v* and *x*, (**e**) ${P}_{gen}$, and (**f**) ${E}_{gen}$.

The TD3 agent of the generator side is trained for 10 s in MATLAB Simulink. The system runs for an additional 40 s, involving states the agent hasn’t encountered during training. The results of the system’s simulation under the effect of irregular sea waves (${H}_{s}= 4 m$ and ${T}_{p}=8 \mathrm{s}$) are depicted in Fig. 10a–f. These include ${F}_{e}$, ${i}_{d}$, ${i}_{q}$, *v* and *x*,${P}_{gen}$, and ${E}_{gen}$. The performance of the PI controllers and the TD3 agent is benchmarked against each other.

The results emphasize that the TD3 agent generator-side controller and the PI controllers achieve the control objectives of reducing the currents’ errors. ${i}_{d}$ is fluctuating around zero and ${i}_{q}$is tracked by the TD3 agent to maximize the energy production from sea waves. Additionally, the following table provides the quantitative comparison between the TD3 agent’s performance and the PI controllers:

**Table 6:** Generator-side TD3 comparison with classical PI controllers.

Metric	PI	TD3 agent	Best
Integral squared error (ISE) of ${i}_{d}$	0.5796	0.434	TD3 agent
ISE of ${i}_{q}$	1.584	0.426	TD3 agent
Average stator losses	3.912 kW	3.946 kW	PI controllers
Average generated power	141.2 kW	141.54 kW	TD3 agent
Energy produced by the generator (${E}_{gen}$)	7.058 MJ	7.077 MJ	TD3 agent

**Fig. 11:**
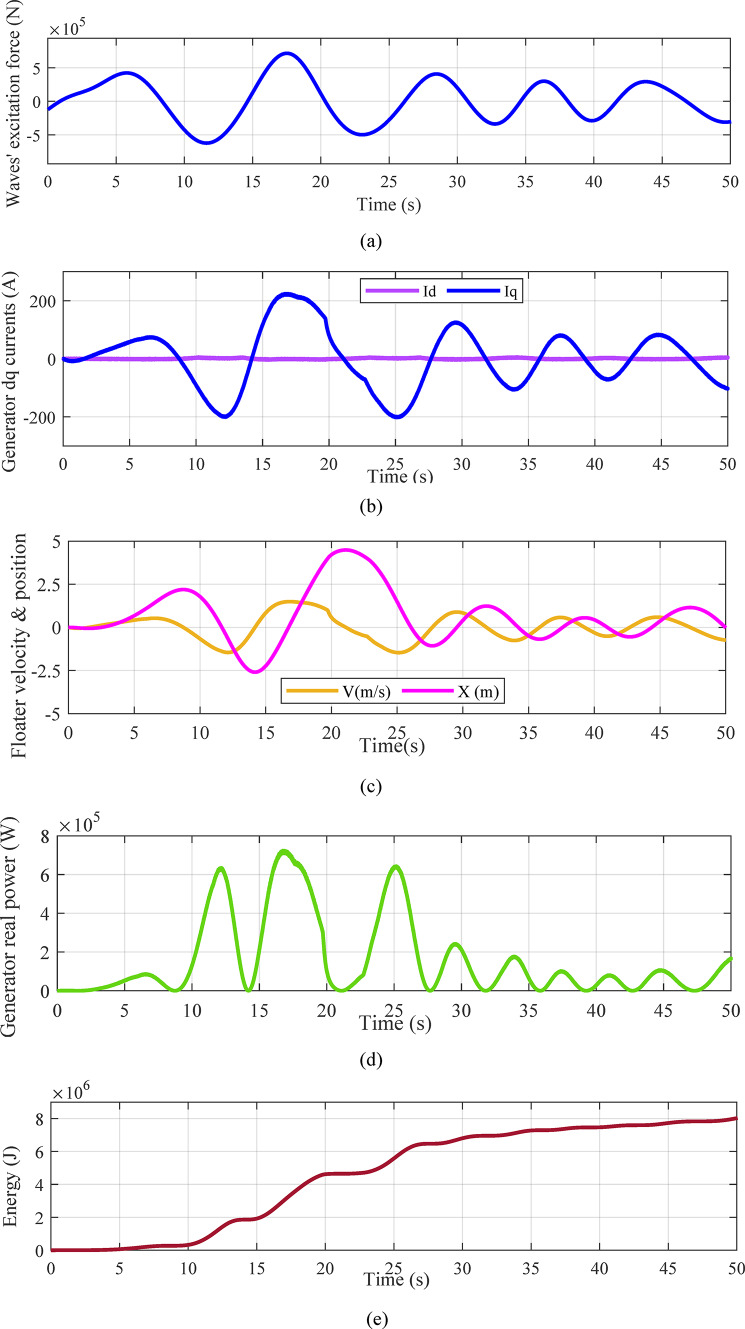
The TD3 agent performance under irregular waves characterized by ${H}_{s}= 2.1 m$ and ${T}_{p}=12 \mathrm{s}$, (**a**) ${F}_{e}$, (**b**) ${i}_{d}$ and ${i}_{q}$, (**c**) *v* and *x*, (**d**) ${P}_{gen}$, and (**e**) ${E}_{gen}$.

**Fig. 12:**
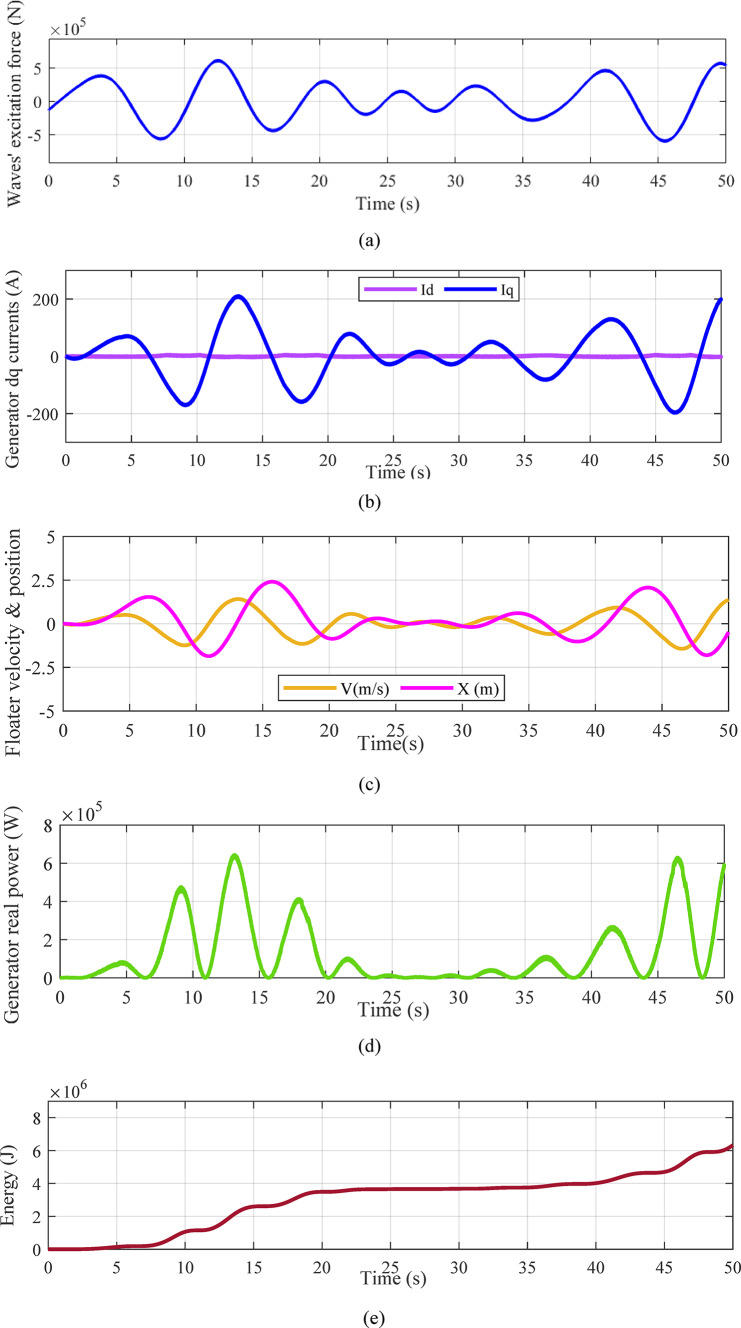
The TD3 agent’s performance under irregular waves characterized by ${H}_{s}= 2.53 m$ and ${T}_{p}=8.58 \mathrm{s}$, (**a**) ${F}_{e}$, (**b**) ${i}_{d}$ and ${i}_{q}$, (**c**) *v* and *x*, (**d**) ${P}_{gen}$, and (**e**) ${E}_{gen}$.

**Fig. 13:**
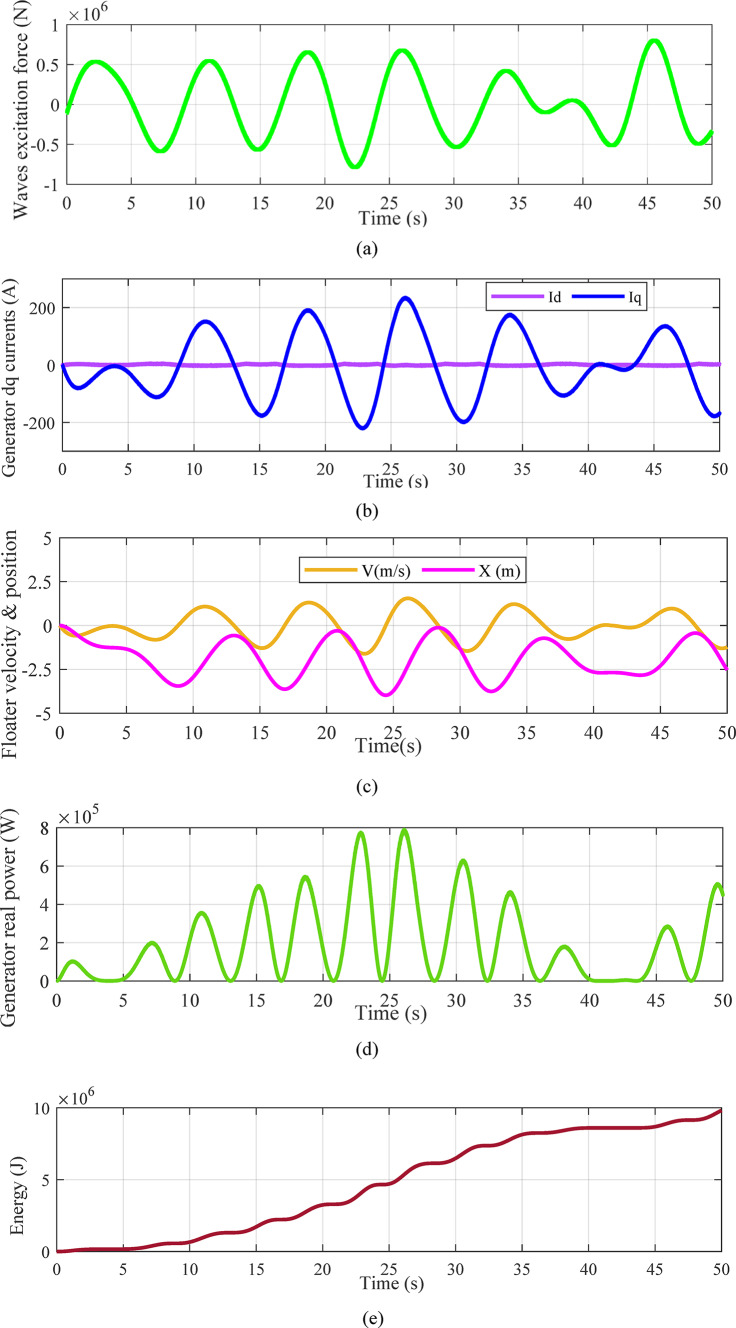
The TD3 agent performance under irregular waves characterized by ${H}_{s}= 4 m$ and ${T}_{p}=8 \mathrm{s}$ and 20% increase in the floater mass (**a**) ${F}_{e}$, (**b**) ${i}_{d}$ and ${i}_{q}$, (**c**) *v* and *x*, (**d**) ${P}_{gen}$, and (**e**) ${E}_{gen}$.

Table 6 confirms the higher control efficiency of the TD3 agent compared to the PI controllers. The ISE for the *dq* currents is lower, and the average generated power and energy over 50 s are higher with the TD3 agent. It should be noted that the TD3 agent has stator losses that are 0.034 kW higher than those of the PI controllers. However, the TD3 agent’s average generated power is 0.34 kW higher. To further confirm the TD3 agent’s tracking reliability, the control efficacy was investigated across multiple sea wave states and with a 20% increase in the floater’s mass (to simulate biofouling). The results are given in Figs. 11, 12 and 13. Additionally, Table 7 provides the ISE for the *dq* currents under these scenarios.

**Table 7:** Generator-side TD3 agent - *dq* currents ISE under different conditions.

Metric	${i}_{d}$ - ISE	${i}_{q}$ - ISE
Irregular waves characterized by ${H}_{s}= 2.1 m$ and ${T}_{p}=12 \mathrm{s}$	0.746	2.64
Irregular waves characterized by ${H}_{s}= 2.53 m$ and ${T}_{p}=8.58 \mathrm{s}$	0.69	1.06
20% increase in the floater mass	0.975	2.055

Table 7 shows that the TD3 agent maintained the ISE of the currents within reasonable values across different sea states, including the scenario of increasing the floater mass due to the attachment of marine organisms to the AWS floater.

### Grid-side agent performance under steady state

**Fig. 14:**
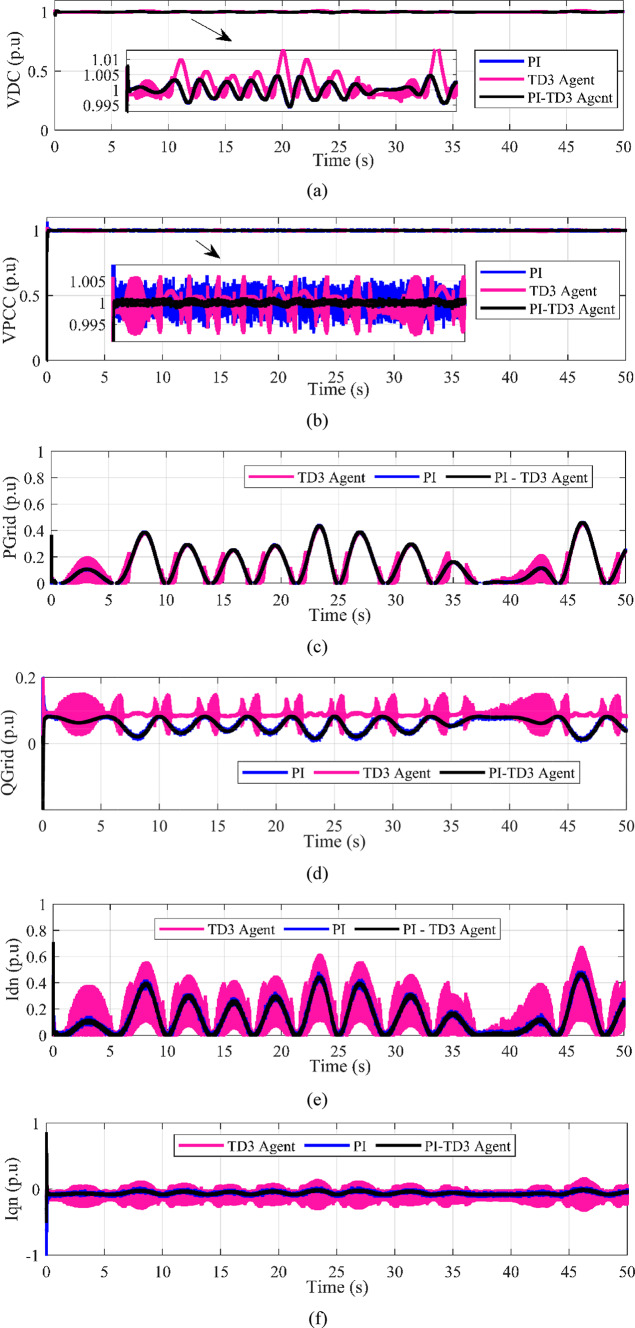
(**a**) $${V}_{DC}$$, (**b**) $${V}_{PCC}$$, (**c**) $${P}_{grid}$$, (**d**) $${Q}_{grid}$$, (**e**) $${i}_{dn}$$, and (**f**) $${i}_{qn}$$.

The TD3 agent and the hybrid-PI agent are trained for 10 s. The simulation runs for another 40 s to evaluate their performance under untrained states. It is benchmarked against the classical PI configuration. The simulation results include ${V}_{DC}$, ${V}_{PCC}$, grid active and reactive powers (${P}_{grid}$and${Q}_{grid}$), and grid-side *dq* currents, as shown in Fig. 14a–f. Furthermore, Table 8 provides a comparison between${ V}_{PCC}$, ${V}_{DC}$, ${I}_{dn}$, and ${I}_{qn}$results obtained in the steady state condition. These are the primary objectives of the various control loops.

**Table 8:** Steady state results comparison.

Metric	PI	TD3 agent	Hybrid PI-TD3	Best
${V}_{PCC}$ – ISE	4.4382	3.524	3.73	TD3 agent followed by the hybrid PI-TD3
${V}_{DC}$ – ISE	0.256	0.865	0.2654	PI controllers and hybrid PI-TD3
${V}_{PCC}$ – peak-to-peak ripple	0.012 pu	0.012 pu	${10}^{-3}$ pu	Hybrid PI-TD3
${V}_{DC}$ – peak-to-peak ripple	0.0105 pu	0.017 pu	0.01 pu	Hybrid PI-TD3 and PI controllers
${V}_{PCC}$ – mean value	0.9998	0.9995	0.9998	TD3 agent
${V}_{DC}$ – mean value	1.0001	1.0016	1.0001	Hybrid PI-TD3 and PI controllers
${V}_{PCC}$ – settling time (5%)	0.042 s	0.02 s	0.06 s	TD3 agent
${V}_{PCC}$ – standard deviation value	0.0094	0.0084	0.0086	TD3 agent
${V}_{DC}$ – standard deviation value	0.0023	0.0038	0.0023	Hybrid PI-TD3 and PI controllers
${I}_{dn}$ – ISE	23.355	Not applicable	5.6802	Hybrid PI-TD3
${I}_{qn}$ – ISE	23.58	Not applicable	8.1842	Hybrid PI-TD3

The steady-state results emphasize that the three controllers were able to stabilize ${V}_{DC}$ and ${V}_{PCC}$ around 1 pu. However, in Fig. 14b, the hybrid PI-TD3 agent has the lowest fluctuations for ${V}_{PCC}$compared with others. Furthermore, the single TD3-agent approach is considered aggressive when controlling these voltages, resulting in waveform fluctuations. This requires a strong filter to remove those switching harmonics. However, this aggressive behavior leads to the minimum settling time for ${V}_{PCC}$. Additionally, the hybrid-PI TD3 offers the best-balanced performance in terms of ISE of ${V}_{PCC}$and${ V}_{DC}$ + the lowest peak-peak ripple for ${V}_{PCC}$.

### Grid-side agent performance under transient state

**Fig. 15:**
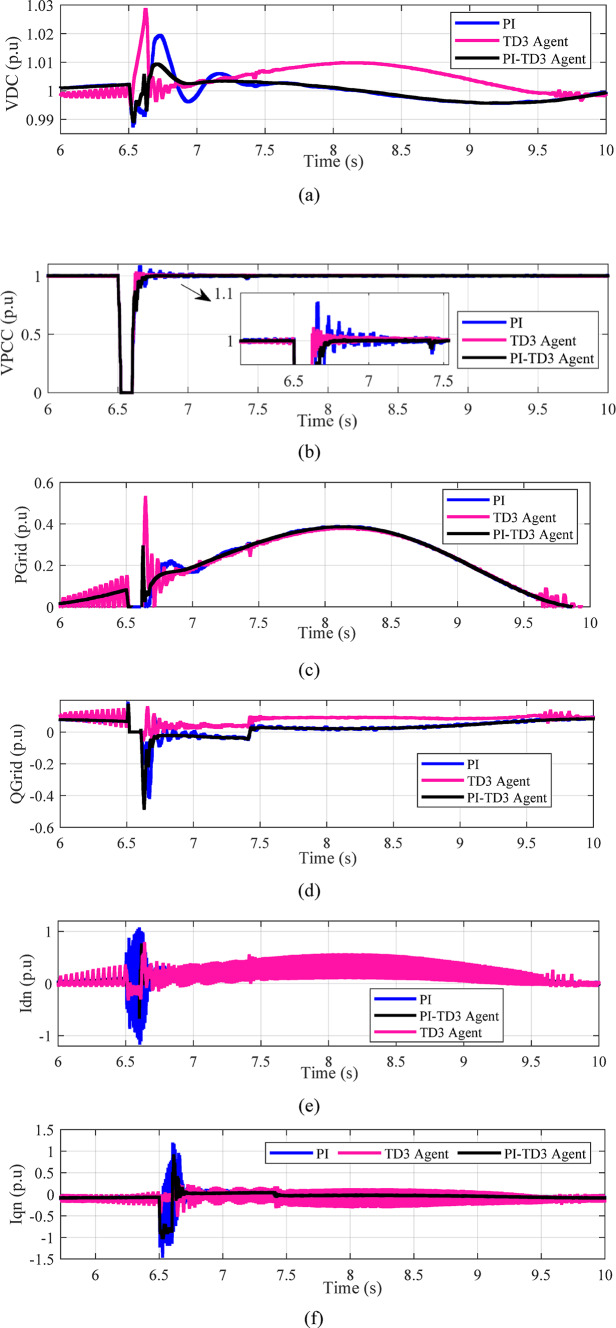
System dynamic response under a symmetrical LLLG fault. The subplots (**a**–**f**) show that the proposed TD3 controllers maintained the DC-link voltage, PCC voltage, and grid currents within stable limits during the fault.

**Fig. 16:**
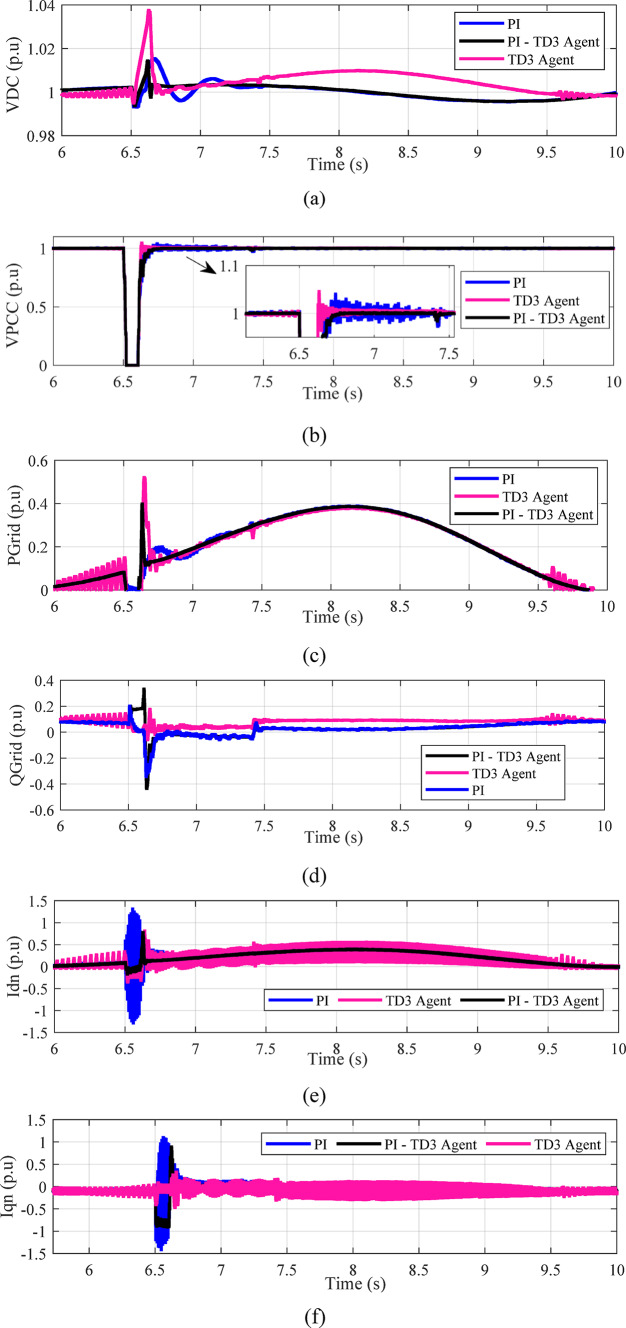
Performance comparison during an asymmetrical 2LG fault. The results illustrate the robustness of the TD3 agent in handling unbalanced conditions compared to the PI controllers.

**Fig. 17:**
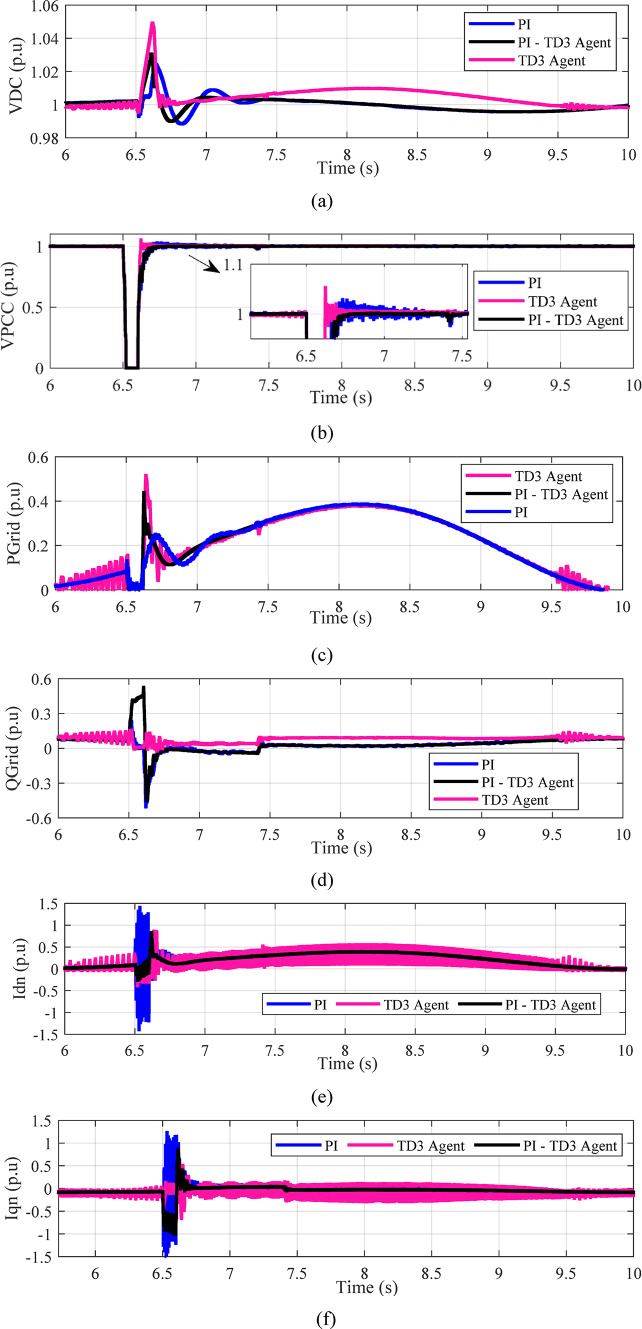
Control effectiveness under an LL fault scenario. The subplots confirm that the *dq* grid currents didn’t reach saturation limits during the disturbance in the case of the TD3 agents.

**Fig. 18:**
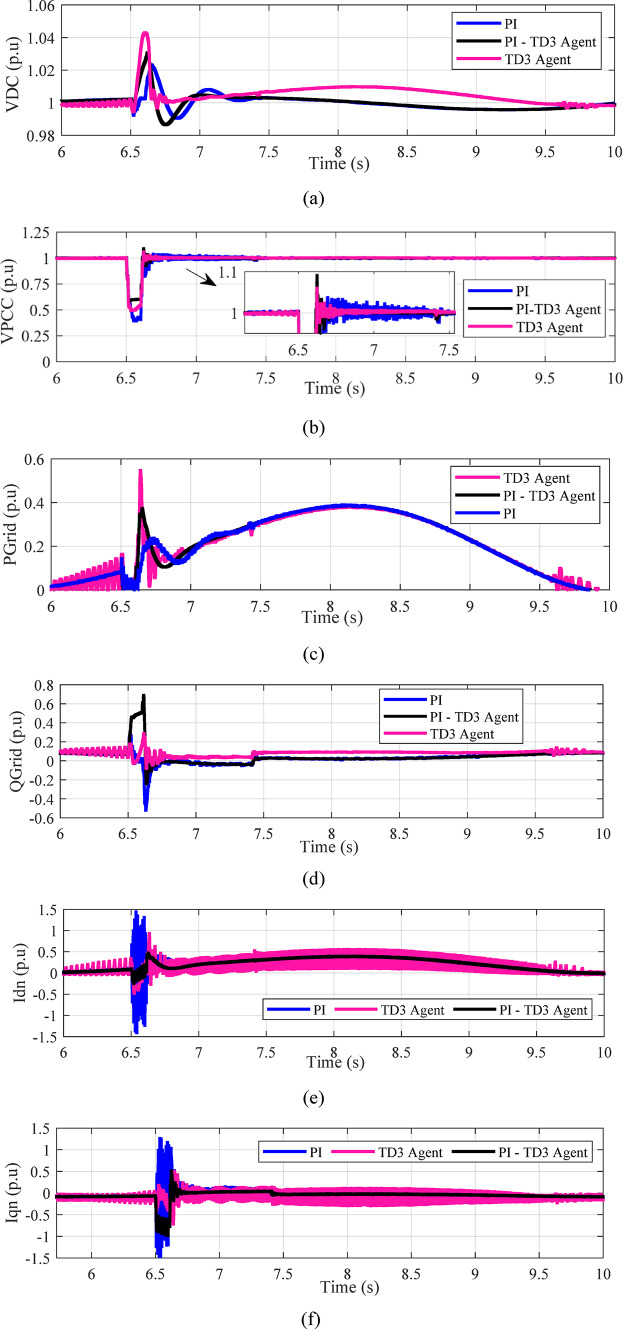
Transient behavior under an LG fault at the PCC. The rapid recovery of all system variables (**a**–**f**) confirms the effectiveness of the TD3 control policy.

**Fig. 19:**
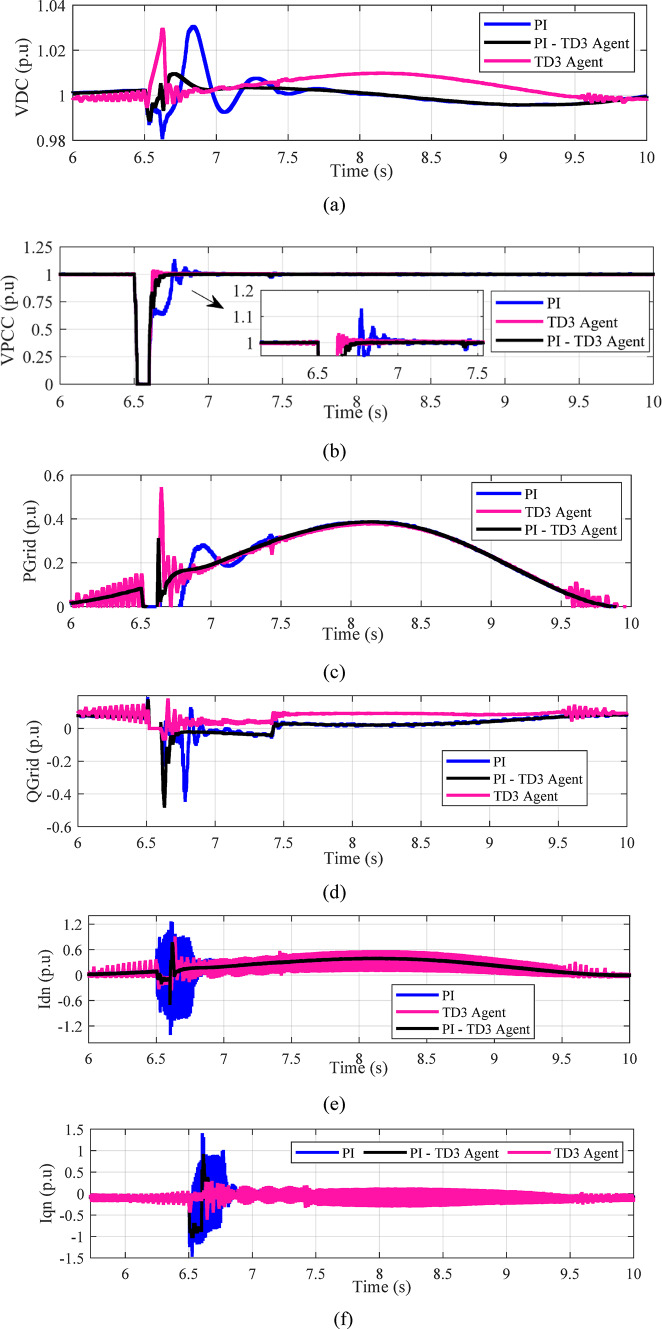
Impact of an LLL fault on system stability. The TD3 agents ensured that active/reactive power, *dq* currents, ${V}_{DC}$, and ${V}_{PCC}$ returned to steady state quickly.

The system is exposed to different grid faults like LLLG (three-line to ground fault), 2LG (two phases A and B + ground fault), LL (line to line fault between phase A and phase B), LG (phase A with ground fault), and LLL (three-line to line fault) faults to assess the performance of the PI controllers, TD3 agent, and the hybrid PI–TD3 agent during these transient states. These faults were examined at point “F” in Fig. 1. Each fault occurs at one of the parallel transmission lines at *t=6.5 s*. The faults are cleared by the circuit breakers associated with the transmission line at *t=6.6 s*. The breakers successfully reclosed at *t=7.4 s*. The fault resistance, grounding resistance, and breaker resistance are 0.1${\Omega }$, 0.01 ${\Omega }$, and 0.01 ${\Omega }$, respectively. The breaker logic complies with the LVRT requirements. The results indicate that breaker trips within 100 ms because ${V}_{PCC}$ drops below 0.5 pu in 99% of scenarios during the various grid faults. The system responses include ${V}_{DC}$, ${V}_{PCC}$, ${P}_{grid}$and${Q}_{grid}$, and grid-side *dq* currents in Figs. 15, 16, 17, 18 and 19. Finally, a quantitative analysis between ${V}_{DC}$, ${V}_{PCC}$, ${P}_{grid}$,${Q}_{grid}$, and the grid-side *dq* currents are specified in Table 9.

**Table 9:** Comparison between the controllers’ results.

Metric	PI	TD3 agent	Hybrid PI-TD3	Comment
${V}_{PCC}$ –maximum overshot and minimum voltage values	LLLG: 1.08 & 0. 2LG: 1.04 & 0. LL: 1.03 & 0. LG: 1.04 & 0.4. LLL: 1.12 & 0.	LLLG: 1.025 & 0. 2LG: 1.046 & 0. LL: 1.053 & 0. LG: 1.06 & 0.5. LLL: 1.03 & 0.	LLLG: 1.001 & 0. 2LG: 1.002 & 0. LL: 1.001 & 0. LG: 1.09 & 0.56. LLL: 1.003 & 0.	Hybrid PI-TD3 has the lowest maximum overshoot in most of the scenarios.
${V}_{DC}$ – maximum overshot and undershot voltage values	LLLG: 1.02 & 0.988. 2LG: 1.015 & 0.993. LL: 1.024 & 0.988. LG: 1.023 & 0.99. LLL: 1.03 & 0.981.	LLLG: 1.028 & 0.996. 2LG: 1.037 & 0.995. LL: 1.049 & 0.995. LG: 1.043 & 0.995. LLL: 1.029 & 0.995.	LLLG: 1.009 & 0.99. 2LG: 1.014 & 0.994. LL: 1.03 & 0.99. LG: 1.03 & 0.987. LLL: 1.01 & 0.989.	TD3 agent has the lowest undershoot. Hybrid PI-TD3 has the lowest overshoot.
${P}_{grid}$ - peak	LLLG: 0.22. 2LG: 0.2. LL: 0.25. LG: 0.237. LLL: 0.28.	LLLG: 0.53. 2LG: 0.52. LL: 0.52. LG: 0.55. LLL: 0.54.	LLLG: 0.29. 2LG: 0.4. LL: 0.44. LG: 0.37. LLL: 0.3.	PI controller has the lowest peak of ${P}_{grid}$ during fault.
${Q}_{grid}$ – max. and min. values	LLLG: 0.183 & -0.41. 2LG: 0.03 & -0.34. LL: 0.233 & -0.5. LG: 0.27 & -0.52. LLL: 0.184 & -0.44.	LLLG: 0.11 & -0.05. 2LG: 0.18 & -0.06. LL: 0.16 & -0.01. LG: 0.29 & -0.07. LLL: 0.17 & -0.06.	LLLG: 0.17 & -0.48. 2LG: 0.33 & -0.43. LL: 0.53 & -0.45. LG: 0.69 & -0.24. LLL: 0.17 & -0.48.	Hybrid PI-TD3 and provides the highest reactive power during fault. However, the TD3 agent has the lowest absorption of reactive power from the grid.
Integral absolute error (IAE) for ${V}_{PCC}$&${V}_{DC}$	LLLG: 158.91. 2LG: 155.04. LL: 151.22. LG: 108.16. LLL: 178.88.	LLLG: 151.24. 2LG: 156.16. LL: 150.97. LG: 104.67. LLL: 151.82.	LLLG: 133.45. 2LG: 136.75. LL: 135.87. LG: 75.55. LLL: 133.63.	Hybrid PI-TD3 has the lowest IAE for the control objectives.
Absolute peak values for ${I}_{dn} \& {I}_{qn}$ $\left(pu\right)$and the peak true RMS current of phase A (*pu*)	LLLG: 1.14, 1.16, 1.22. 2LG: 1.311, 1.41, 2.54. LL: 1.4, 1.23, 1.39. LG: 1.44, 1.5, 3.49. LLL: 1.37, 1.44, 1.28.	LLLG: 0.771, 0.5, 0.489. 2LG: 0.81, 0.46, 3.15. LL: 0.86, 0.66, 0.83. LG: 0.93, 0.711, 2.42. LLL: 0.876, 0.575, 0.528.	LLLG: 0.714, 1, 0.99. 2LG: 0.77, 0.89, 3.477. LL: 0.832, 0.996, 0.828. LG: 0.47, 0.969, 2.78. LLL: 0.74, 1, 0.994.	Hybrid PI-TD3 and TD3 agent maintain the *dq* currents in the [-1 1] range. Unlike the PI controllers that exceed 1 pu by a large margin during multiple scenarios. The single TD3 agent has the minimum RMS value of phase A in all faults except in 2LG fault.
Settling time (2%) - ${V}_{PCC}$	LLLG: 0.273 s. 2LG: 0.213 s. LL: 0.08 s. LG: 0.4 s. LLL: 0.263 s.	LLLG: 0.058 s. 2LG: 0.05 s. LL: 0.024 s. LG: 0.072 s. LLL: 0.058 s.	LLLG: 0.099 s. 2LG: 0.084 s. LL: 0.1 s. LG: 0.1 s. LLL: 0.1 s.	TD3 agent has the lowest settling time followed by the hybrid PI-TD3 agent.
Settling time (2%) - ${V}_{DC}$	LLLG: - 2LG: - LL: 0.069 s. LG: 0.074 s. LLL: 0.3 s.	LLLG: 0.038 s. 2LG: 0.046 s. LL: 0.042 s. LG: 0.037 s. LLL: 0.039 s.	LLLG: - 2LG: - LL: 0.022 s. LG: 0.044 s. LLL: -	The hybrid PI-TD3 maintained the voltage within 2% even during fault conditions in most cases, followed by the PI controllers. Despite,${V}_{DC}$exceeded 2% several times in the case of the single TD3 agent, the agent was able to quickly bring it back to the 2% range.
Post-fault recovery time - ${V}_{PCC}$	LLLG: 0.042 s. 2LG: 0.056 s. LL: 0.052 s. LG: 0.015 s. LLL: 0.152 s.	LLLG: 0.02 s. 2LG: 0.023 s. LL: 0.015 s. LG: 0.014 s. LLL: 0.021 s.	LLLG: 0.042 s. 2LG: 0.05 s. LL: 0.042 s. LG: 0.013 s. LLL: 0.042 s.	TD3 agent has the quickest recovery of ${V}_{PCC}$ compared to others.

Under different fault conditions, grid-side *dq* currents are successfully suppressed under 1 pu by both the TD3 agent and the hybrid PI-TD3 agent. In contrast, the classical PI controllers failed to keep them below 1 pu, and in many cases, the currents reached 1.5 pu. The hybrid PI-TD3 and TD3 agents are considered the most effective two controllers compared to the classical PI controllers. The hybrid PI-TD3 has almost no overshoot in ${V}_{PCC}$and the lowest overshoot in ${V}_{DC}$during fault conditions. Second, the TD3 agent has the lowest undershoot in ${V}_{DC}$. While the PI control system has the lowest overshoot for the provided grid-active power under fault conditions. However, we are more concerned with providing reactive power. That’s why the hybrid PI-TD3 outperformed the other controllers, as it has the highest provided ${Q}_{grid}$ in most cases of grid faults. In summary, the suggested approach is a hybrid one that combines the TD3 agent with PI controllers to achieve optimal performance.

### Testing the hybrid PI-TD3 grid-side agent under different grid short circuit ratios (SCR)

**Fig. 20:**
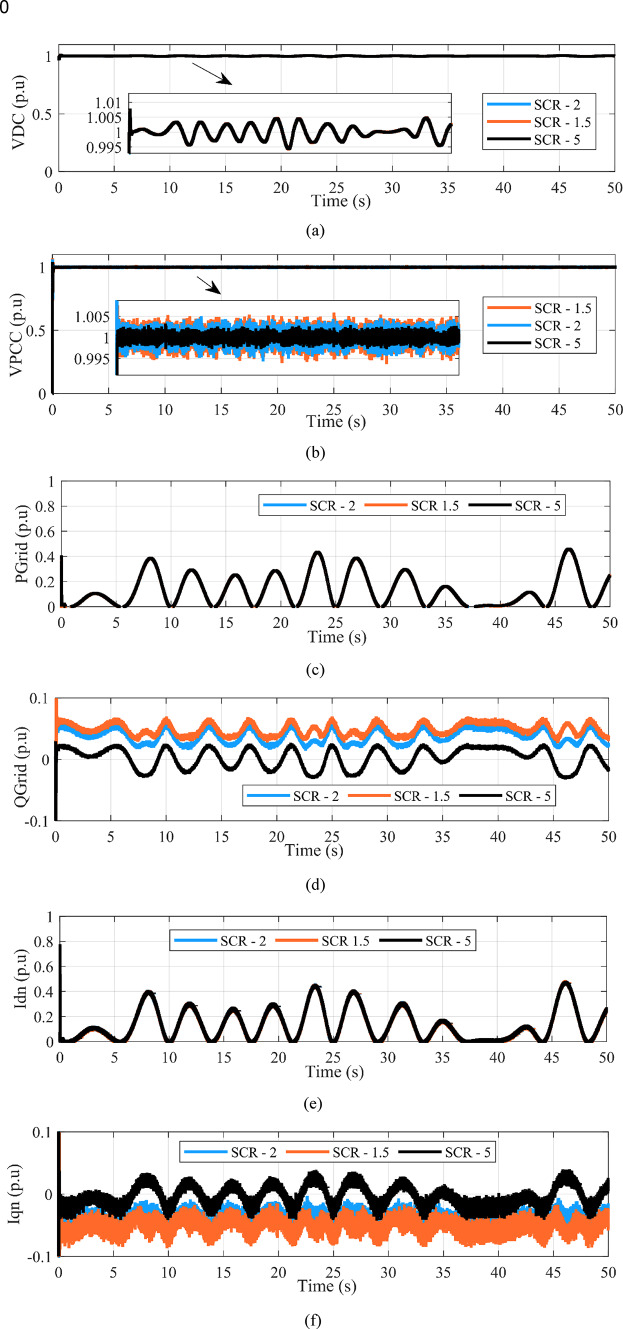
Impact of the grid SCR on the hybrid PI-TD3 agent performance.

**Fig. 21:**
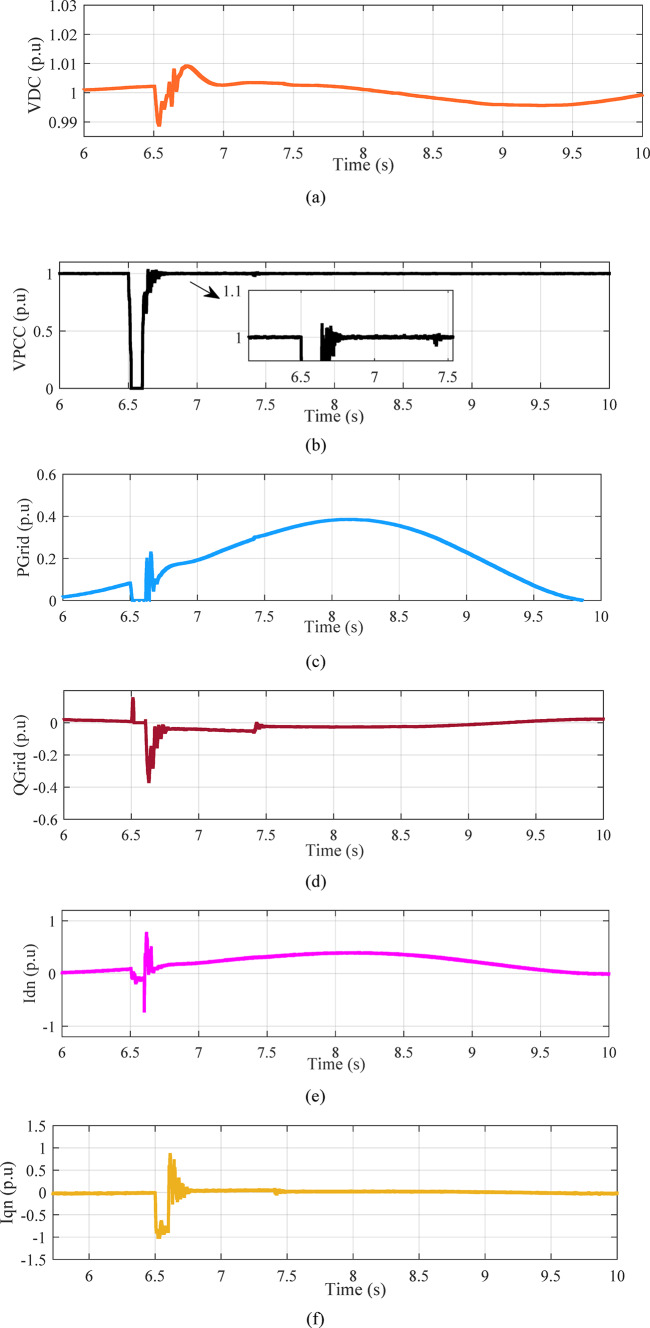
The system response during an LLLG fault under grid with SCR = 5.

In this part, the performance of the hybrid PI-TD3 agent was investigated under different grid SCR ratios (1.5, 2, and 5). The SCR is the ratio between the short circuit MVA of the grid with respect to the rated generator power (1 MVA). This evaluates the behavior of the grid-connected system under weak and moderate power grids. The waveforms of the systems under steady-state are given in Fig. 20. Moreover, the system was also assessed under the effect of an LLLG fault in Fig. 21.

The results emphasize that the system can survive extreme conditions (SCR = 1.5). However, under lower values like SCR = 1, the system wouldn’t be able to keep ${V}_{PCC}$ or ${V}_{DC}$ at 1 pu. Moreover, Fig. 20d shows that as the grid becomes weaker (lower SCR), the inverter injects more reactive power to keep ${V}_{PCC}$at its nominal value. During a LLLG in a grid with SCR = 5, the TD3 controller achieved the control objectives as before. However, the TD3 agent needs to be trained during the transient state in case the controller is installed in a weak grid with SCR lower than 2 to prevent high overshooting in ${V}_{PCC}$.

## Conclusions

This work presents a novel approach using two twin-delayed deep learning agents to improve the performance of a grid-connected WEC. A TD3 agent is deployed on the generator side to reduce stator electrical losses and increase energy production from sea waves. This agent was compared with the classical PI controller configuration and achieved lower ISE for the *dq* currents. Moreover, the agent was subjected to various sea states and to changes in the floater mass to assess its reliability. On the grid side, two TD3-agent methodologies are developed. The first is the replacement of the classical cascaded PI control loop (four PI controllers) with a TD3 agent that takes multiple observations as input and provides the grid-side *dq* voltages as control actions. In the second approach, a hybrid PI-TD3 agent is deployed. In this control strategy, the first two PI controllers that provide the reference grid *dq* currents are kept as they are, and the second pair of PI controllers is exchanged with a TD3 agent. The two grid-side agents and the PI controllers are benchmarked against each other under the steady-state for 50 s. In addition, the three configurations are analyzed during transient conditions under different grid faults to evaluate the agents’ performance. After evaluating the results, the most effective control strategy is the hybrid approach, which combines the TD3 agent with PI controllers. This combination provides the greatest performance in both steady and transient states. To further confirm the hybrid approach’s reliability, the controller’s effectiveness was analyzed at different SCR values (1.5, 3, 5). The controller demonstrated its efficiency in steady-state and transient-state conditions (SCR = 5), further emphasizing its superior performance.

## Data Availability

The datasets generated and/or analyzed during the current study are available from the corresponding author on reasonable request.
